# Role of human papillomavirus status after conization for high‐grade cervical intraepithelial neoplasia

**DOI:** 10.1002/ijc.33251

**Published:** 2020-09-01

**Authors:** Huei‐Jean Huang, Hsiu‐Jung Tung, Lan‐Yan Yang, Angel Chao, Yun‐Hsin Tang, Hung‐Hsueh Chou, Wei‐Yang Chang, Ren‐Chin Wu, Chu‐Chun Huang, Chiao‐Yun Lin, Min‐Jie Liao, Wei‐Chun Chen, Cheng‐Tao Lin, Min‐Yu Chen, Kuan‐Gen Huang, Chin‐Jung Wang, Ting‐Chang Chang, Chyong‐Huey Lai

**Affiliations:** ^1^ Department of Obstetrics and Gynecology Chang Gung Memorial Hospital, Linkou Branch and Chang Gung University Taoyuan Taiwan; ^2^ Gynecologic Cancer Research Center, Chang Gung Memorial Hospital Taoyuan Taiwan; ^3^ Clinical Trial Center Chang Gung Memorial Hospital, Linkou Branch Taoyuan Taiwan; ^4^ Department of Pathology Chang Gung Memorial Hospital, Linkou Branch and Chang Gung University Taoyuan Taiwan

**Keywords:** cervical intraepithelial neoplasia, conization, human papillomavirus, recurrence

## Abstract

Human papillomavirus (HPV) is the well‐established etiologic factor for cervical neoplasia. Cervical conization constitutes an effective treatment for high‐grade cervical intraepithelial neoplasia (HG‐CIN). We conducted an observational study for long‐term outcomes and HPV genotype changes after conization for HG‐CIN. Between 2008 and 2014, patients with newly diagnosed HG‐CIN before conization (surveillance new [SN] group) and those who had undergone conization without hysterectomy (surveillance previous [SP] group) were enrolled. HPV testing and Pap smear were performed periodically for the SN and SP (collectively S) groups. All other patients receiving conization for HG‐CIN during the study period were identified from our hospital database. Those eligible but not enrolled into our study were assigned to the non‐surveillance (non‐S) group. For the S group (n = 493), the median follow‐up period was 74.3 months. Eighty‐four cases had recurrent CIN Grade 2 or worse (CIN2+) (5‐year cumulative rate: 14.8%), of which six had invasive cancer. Among the 84 patients, 65 (77.4%) exhibited type‐specific persistence in the paired HPV results, whereas only 7 (8.3%) harbored new HPV types that belonged to the 9‐valent vaccine types. Among the 7397 non‐S patients, 789 demonstrated recurrent CIN2+, of which 57 had invasive cancer. The stages distribution of those progressed to invasive cancer in the non‐S group were more advanced than the S group (*P* = .033). Active surveillance might reduce the severity of those progressed to cancer. Because a majority of the patients with recurrent CIN2+ had persistent type‐specific HPV infections, effective therapeutic vaccines are an unmet medical need.

## Abstract 2

**What's new?**

High‐grade cervical intraepithelial neoplasia (HG‐CIN), a cervical carcinoma precursor, frequently is caused by high‐risk human papillomavirus (hr‐HPV) infection. HG‐CIN can be treated by cervical conization, though the procedure does not eradicate hr‐HPV, potentially enabling CIN recurrence. Here, in Taiwanese patients, investigation of HPV genotype changes in relation to HG‐CIN status after conization shows that 77.4 percent of patients with recurrent CIN 2 grade or worse (CIN2+) after conization had persistent type‐specific HPV infections. Vaccination against the remaining high‐risk HPVs prevented recurrent CIN2+ in only 8.3 percent of patients, revealing an unmet need for effective therapeutic vaccines.

**Abbreviations**
ASCCP: American Society for Colposcopy and Cervical Pathology
CI: confidence interval
CIN2+: CIN Grade 2 or worse
FFPE: formalin‐fixed paraffin‐embedded
FIGO: Federation of Gynecology and Obstetrics
HG‐CIN: high‐grade cervical intraepithelial neoplasia
HPV: human papillomavirus
hr‐HPV: high‐risk HPV
HRs: hazard ratios
LEEP: loop electrical excision procedure
lr‐HPV: low‐risk HPV
non‐S: non‐surveillance
SN: surveillance new
SP: surveillance previous

## 1 INTRODUCTION

Cervical carcinoma is a multistep slow‐developing disease and is often preceded by high‐grade cervical intraepithelial neoplasia (HG‐CIN); human papillomavirus (HPV) is the well‐established causative agent of HG‐CIN.^1^ Overall HPV prevalence rates in patients treated for HG‐CIN ranged from 84.9% to 98.5%, and that in cervical carcinoma ranged from 89.7% to 96.6%.^2^, ^3^, ^4^ More than 228 HPV types have been molecularly identified, of which 40 can spread through genital contact.^1^, ^2^, ^5^ A total of 15 HPV types are classified as high‐risk HPV (hr‐HPV) associated with cervical carcinoma, including HPV16, 18, 31, 33, 35, 39, 45, 51, 52, 56, 58, 59, 68, 73, and 82 (MM4). Additionally, 3 types are grouped as probable hr‐HPV types (26, 53, and 66), and 12 are classified as low‐risk HPV (lr‐HPV) types (6, 11, 40, 42, 43, 44, 54, 61, 70, 72, 81, and CP6108).^1^

Data on HPV distribution in HG‐CIN and cervical carcinoma patients are essential for understanding the natural history of HPV‐associated cervical lesions. HPV16 has consistently been the predominant HPV type worldwide, both in HG‐CIN and cervical carcinoma patients. However, its absolute prevalence and the distribution of other genotypes vary in different countries and ethnic groups.^2^, ^3^, ^4^, ^6^, ^7^ A meta‐analysis in 7094 HG‐CIN patients demonstrated that HPV16, 31, 33, 58, 18, 52, 35, and 51 were the most prevalent.^3^ Our previous study of 1086 samples indicated HPV16, 52, and 58^8^ as the prevalent HPV types, consistent with the results of a prospective study across five Asian countries.^9^

Cervical conization constitutes an effective therapeutic treatment for HG‐CIN and early invasive cancer; however, this treatment cannot eradicate hr‐HPV completely. Wide‐ranging recurrence rates of CIN after conization of 0.35% to 69% were reported.^10^ HPV follow‐up status, margin status, and follow‐up cervical cytology were significant predictors of residual/recurrent HG‐CIN.^10^ Patients who received treatment for HG‐CIN demonstrated increased risk of invasive cervical cancer compared to the general population for at least 10 years.^11^ A recent systemic review reported the median values of non–type‐specific HPV persistence after treatment for HG‐CIN as 27% at 3 months, 21% at 6 months, 15% at 12 months, and 10% at 24 months.^12^ Increasing evidence has supported the role of HPV testing in post‐conization surveillance strategies. However, there is insufficient evidence from randomized clinical trials, and long‐term follow‐up HPV genotype data are lacking.^11^

In our study, we evaluated a post‐conization surveillance strategy involving follow‐up of Pap and HPV status of HG‐CIN patients treated with conization to delineate HPV subtype changes, and we compared the cumulative recurrence/progression rate in these patients with that in usual‐care patients.

## 2 MATERIALS AND METHODS

### 2.1 Study design

We conducted a prospective observational study of patients who were newly diagnosed with HG‐CIN before conization (surveillance new [SN] group) and those who had received conization without hysterectomy (surveillance previous [SP] group) at Chang Gung Memorial Hospital and were willing to participate from the point of signing an informed consent form. Conization with the loop electrical excision procedure (LEEP) was most frequently performed, and cold knife or laser excision was chosen occasionally depending on the individual clinical scenario. For the SN group, HPV testing was performed before conization. For all surveillance (SN + SP; S group hereafter) patients, cervical and vaginal cytology and HPV testing were performed every 6 months (except that the first post‐conization visit could be conducted between 3 and 6 months) and every year for those who had no events (abnormal Pap/histology or HPV‐positive results) in the 5 years after conization. Those with HPV‐positive results or abnormal Pap smears underwent colposcopy and directed biopsy, as indicated. For SP group patients, the follow‐up method in non‐S group was cytology alone 6 to 12 months. Their cytology/pathology results (all SP group) as well as HPV testing results (some of them had HPV tests) before enrollment were retrieved from our hospital database. HG‐CIN events in the SP group after initial conization and before enrollment were recorded as a recurrent HG‐CIN event, and long‐term outcomes were analyzed. All the S group patients gave their written consent, and the study was approved by the institutional review board (IRB97‐1702A3, 98‐0466A3, 100‐2900A3, and 20170071B0).

Total HPV clearance was defined as HPV‐negative results throughout post‐conization follow‐up. Subsequent total clearance was defined as HPV‐negative results in at least two visits by the end of the study, despite HPV‐positive results in earlier post‐conization visit(s). Patients with negative results during follow‐up that turned positive were not considered to have HPV clearance. Type‐specific clearance was defined as negative results for a specific HPV type at two visits without reappearance at subsequent follow‐ups. Time to HPV clearance was defined as the time from initial conization of patients with HPV‐positive HG‐CIN to the first of the two visits with HPV‐negative results. HPV acquisition was defined as detection of HPV after at least two preceding visits with HPV‐negative results.

### 2.2 HPV genotyping

DNA was extracted from formalin‐fixed paraffin‐embedded (FFPE) tissue or swab cervical specimens, as previously described.^4^, ^8^, ^10^, ^13^ SPF1/GP6+ consensus primers were employed to amplify a fragment of approximately 184 bp in the L1 open reading frame. Glyceraldehyde‐3‐phosphate dehydrogenase polymerase chain reaction (PCR) was performed as an internal control. HPV genotyping was performed through HPV blotting by using 15 μL of the resultant amplicons that were subsequently hybridized with an Easychip HPV Blot (King Car, I‐Lan, Taiwan) membrane. A total of 38 types of HPV (6, 11, 16, 18, 26, 31, 32, 33, 35, 37, 39, 42, 43, 44, 45, 51, 52, 53, 54, 55, 56, 58, 59, 61, 62, 66, 67, 68, 69, 70, 71 [CP8061], 72, 74, 81 [CP8304], 82 [MM4], 83 [MM7], 84 [MM8], and L1AE5) were detected in a single reaction, as previously described. The annealing conditions were different for each set of primers. Type‐specific PCR reactions were performed to validate multiple types and HPV‐negativity on HPV blot.^4^, ^8^, ^10^, ^13^ For the remaining HPV‐negative results obtained through type‐specific PCR, HPV16/18/52/58 whole genome amplification generated overlapping amplicons, and direct sequencing was performed (Supplementary Methods, Tables S1‐S4, Figures S1‐S4).

### 2.3 Statistical analysis

In our study, recurrence/progression was defined as diagnosis of HG‐CIN or cervical cancer (CIN Grade 2 or worse [CIN2+]) at least 3 months after the initial diagnosis. Those diagnosed with HG‐CIN within 3 months were considered to have persistent disease. Those diagnosed with invasive cancer within 3 months were considered to have invasive cervical cancer with the initial conization under‐diagnosis. If multiple events occurred in the same patient, the time to recurrence/progression was defined as the time of the first HG‐CIN event. The time‐dependent HPV infection status of the study patients was evaluated from first conization to the date of the last follow‐up visit, progression to cancer, or hysterectomy. Patients with multiple infections were categorized by the order of cervical cancer prevalence (eg, HPV16 and then HPV18).^1^ hr‐HPV types were defined as HPV16,18, 31, 33, 35, 39, 45, 51, 52, 56, 58, 59, and 68,^1^ and probable hr‐HPV types were grouped with lr‐HPV types as lr‐types in our study (6, 11, 26, 32, 37, 42, 43, 44, 53, 54, 55, 61, 62, 66, 67, 69, 70, 71, 72, 74, 81, 82, 83, 84, and L1AE5) on the basis of low prevalence of HPV26, 53, and 66 in patients with HG‐CIN and cervical cancer (Table S5).^4^, ^8^, ^10^, ^13^

**FIGURE 1:**
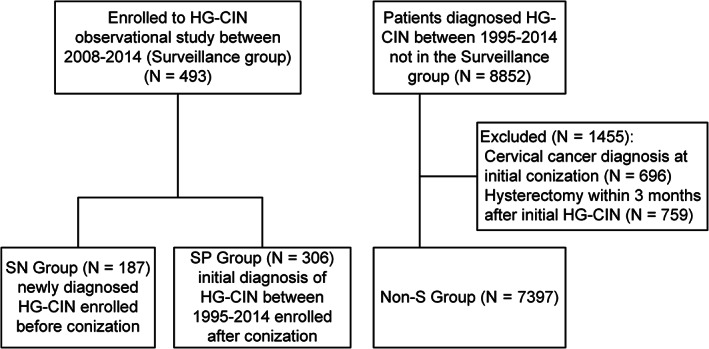
Flow diagram. non‐S: non‐surveillance; SN, surveillance new; SP, surveillance previous

The SP group patients had their earliest diagnosis of initial HG‐CIN in June 1995, and the last S group patient was enrolled in December 2014; therefore, all patients diagnosed with HG‐CIN between June 1995 and December 2014 were identified from the hospital database (Figure 1). Those who had received hysterectomy or were diagnosed with invasive cervical cancer within 3 months after conization were excluded. Stages were reclassified using the 2018 International Federation of Gynecology and Obstetrics (FIGO) staging system for the cases that had progressed to invasive cervical cancer.^14^ The cumulative rates of CIN2+ or invasive cancer in the non‐surveillance (non‐S) group were compared to those in the S group. Archival tissues of both initial HG‐CIN and subsequent cancer were retrieved for HPV genotyping in the non‐S group only for patients who showed progression to invasive cancer.

Between‐group comparisons of cumulative recurrence were based on the Kaplan‐Meier method and log‐rank tests. Pearson's chi‐square test or Fisher's exact test was used to evaluate proportion differences between designated groups. Their hazard ratios (HRs) and 95% confidence intervals (CIs) were calculated. Continuous covariates were compared between groups using both parametric and nonparametric approaches, mainly, the Student *t* and Mann‐Whitney *U* test, as appropriate. Statistical analysis was performed using SAS version 9.4 (Cary, NC). All tests were two‐sided, and a *P* value of <.05 was considered statistically significant.

## 3 RESULTS

A total of 493 patients (SN group: n = 187; SP group: n = 306) were enrolled between June 2008 and December 2014 for this longitudinal surveillance study. A total of 8852 patients who were diagnosed with HG‐CIN between June 1995 and December 2014 were not included in the S group. After excluding patients with cervical cancer diagnosis (n = 696) at initial conization and those who underwent hysterectomy within 3 months after the initial HG‐CIN diagnosis (n = 759), the remaining 7397 patients served as usual‐care controls (ie, non‐S group; Figure 1). Table 1 presents the demographic characteristics of the S group vs the non‐S group and the SN group vs the SP group. The median age of the S group participants was 40.9 years (range: 20.2‐78.0 years), and no significant difference was observed in age between the SN and SP groups. The median follow‐up period of the S group (74.3 months; range: 0‐275.5 months) was significantly longer than that of the non‐S group (19.8 months; range: 0‐276.7 months) (*P* < .001). The median follow‐up period of the SN group (30.5 months; range: 0‐97.5 months) was significantly shorter than that of the SP group (126.1 months; range: 3.5‐275.5 months) (*P* < .001). CIN was more severe in the non‐S group than in the S group (*P* = .019), whereas the difference in the CIN grade or margin status between the SN and SP groups was not significant.

**TABLE 1:** Demographics of cases receiving conization for high‐grade cervical intraepithelial neoplasia between 1995 and 2014

	Non‐S group		S group			SN group		SP group		
	(n = 7397)		(n = 493)			(n = 187)		(n = 306)		
Variable	n	%	n	%	*P* value^a^	n	(%)	n	(%)	*P* value^a^
Age, y (median, range)	NA	NA	40.9	(20.2, 78.0)	NA	39.2	(20.2, 75.4)	41.4	(20.6, 78.0)	.085
Follow‐up time, months (median, range)	19.8	(0, 276.7)	74.3	(0, 275.5)	<.001	30.5	(0, 97.5)	126.1	(3.5, 275.5)	<.001
HPV‐positive at initial conization	NA	NA	465	(94.3)	NA	180	(96.3)	285	(93.1)	.147
Histology					.019					.406
CIN2	3691	(49.9)	273	(55.4)		108	(57.8)	165	(53.9)	
CIN3	3706	(50.1)	220	(44.6)		79	(42.2)	141	(46.1)	
Margin involved					NA					.082
Margin (−)	NA	NA	116	(23.5)		45	(24.1)	71	(23.2)	
Margin (+)	NA	NA	174	(35.3)		76	(40.6)	98	(32.0)	
Margin (not recorded)	NA	NA	203	(41.2)		66	(35.3)	137	(44.8)^b^	

Abbreviations: CIN, cervical intraepithelial neoplasia; NA, not applicable; Non‐S, non‐surveillance; SN, surveillance new; SP, surveillance previous.

^a^ T test, Chi‐square test as appropriate.

^b^ For those with diagnosis before 1999, the electronic pathology reports do not show margin status unless positive.

HPV was detected in 94.3% of the S group participants in the FFPE tissue of HG‐CIN, primary conization specimens or pre‐conization biopsy. The leading HPV types were HPV16 (31.6%), HPV52 (25.8%), HPV58 (15.6%), HPV33 (6.5%), HPV31 (5.7%), and HPV18 (5.5%), whereas 5.7% of the patients were HPV‐negative. Among HPV‐positive patients (n = 465), 97 (20.9%) were affected by multiple HPV types (Table 2). The recurrent CIN2+ rate was significantly higher among HPV‐positive patients in the initial conization samples than in HPV‐negative patients (*P* = .008). The rates of recurrence/progression of probable hr‐HPVs and lr‐HPVs were not different. Additionally, none of the patients had shown progression to cancer (Table S5). The data in our study supported our grouping policy.

**TABLE 2:** Type‐specific HPV recurrence/progression rates in the surveillance group

	HPV at initial conization		Recurrent CIN2+		Progression to invasive cancer	
	n	(%)	n	(%)	n	(%)
Total	493	(100)	84	(17.0)	6	(1.2)
HPV−	28	(5.7)	0	(0)^c^ ^,^*	0	(0)^c^ ^,^**
HPV+	465	(94.3)	84	(18.1)^c^ ^,^*	6	(1.3)^c^ ^,^**
Single	368	(74.6)	62	(16.8)	5	(1.4)
Multiple	97	(19.7)	22	(22.7)	1	(1.0)
HPV16^a^	156	(31.6)	36	(23.1)	4	(2.6)
HPV18^a^	27	(5.5)	4	(14.8)	1	(3.7)
HPV31^a^	28	(5.7)	9	(32.1)	0	(0)
HPV33^a^	32	(6.5)	9	(28.1)	0	(0)
HPV35^a^	6	(1.2)	1	(16.7)	0	(0)
HPV39^a^	13	(2.6)	1	(7.7)	0	(0)
HPV45^a^	5	(1.0)	0	(0)	0	(0)
HPV51^a^	25	(5.1)	3	(12.0)	0	(0)
HPV52^a^	127	(25.8)	19	(15.0)	1	(0.8)
HPV56^a^	12	(2.4)	4	(33.3)	0	(0)
HPV58^a^	77	(15.6)	12	(15.6)	1	(1.3)
HPV59^a^	3	(0.6)	0	(0)	0	(0)
HPV68^a^	6	(1.2)	2	(33.3)	0	(0)
HPV82^a^	7	(1.4)	0	(0)	0	(0)
Lr‐HPVs^b^	55	(11.2)	12	(21.8)	0	(0)

Abbreviation: HPV, human papillomavirus.

^a^ Same woman can be counted more than once because of multiple infections.

^b^ Probable hr‐HPVs (HPV 26, 53, and 66) were grouped with low‐risk types as lr‐HPVs (6, 11, 26, 32, 37, 42, 43, 44, 53, 54, 55, 61, 62, 66, 67, 69, 70, 71, 72, 74, 81, 83, 84, and L1AE5) based on low prevalence of HPV 26, 53, and 66 in HG‐CIN and cervical cancer in Taiwanese data.^4^, ^8^, ^10^, ^13^

^c^ *P* values for comparisons annotated by * and ** are .008 and >.999 respectively, by Fisher's exact test.

In the S group, 84 patients were diagnosed with CIN2+ during follow‐up, that is, a 5‐year cumulative recurrence/progression rate of 14.8% was obtained (SN vs SP: 14.4% vs 16.1%, *P* = .230) (Figure S5). The median time between initial HG‐CIN diagnosis and recurrence/progression was 19.8 months (range: 3.3‐162.2 months), with 23.8% of these patients developing recurrent CIN2+ >5 years after conization. Among them, six patients in the S group developed invasive cervical cancer (five patients had SCCs and one patient had adenocarcinoma of FIGO stages IA1‐IB1 at 1.3, 1.9, 3.1, 7.4, 8.0, and 15.2 years after initial HG‐CIN diagnosis) (Table 2 and Table S6).

**FIGURE 2:**
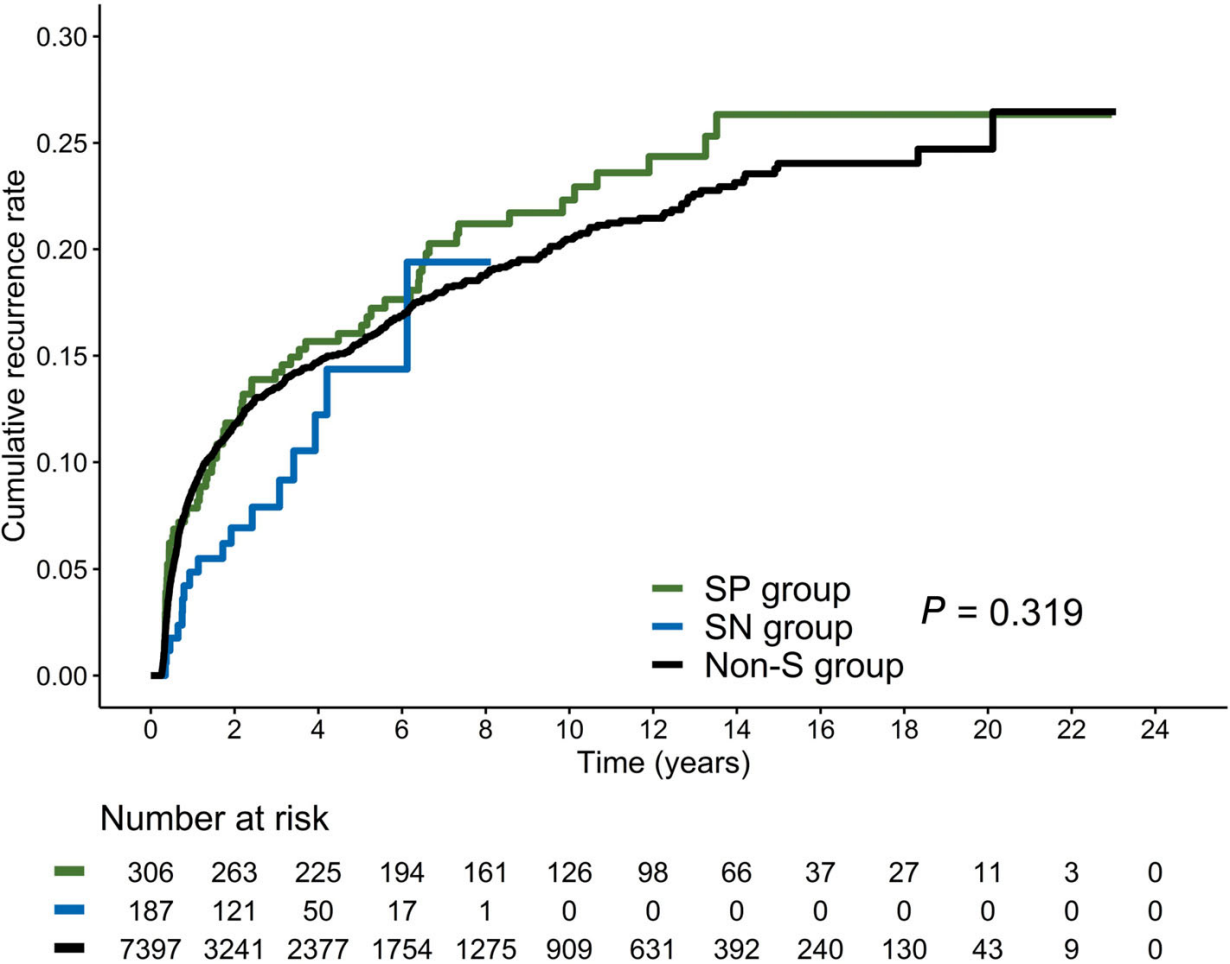
Kaplan‐Meier curves of cumulative recurrence/progression of surveillance new (SN) group, surveillance previous (SP) group, and non‐surveillance (non‐S) group

Among the 7397 non‐S group patients (789 patients had recurrence of CIN2+), 57 had invasive cancer (15 Stage IA1, 5 IA2, 10 IB1, 2 IB2, 1 IB3, 3 IIA1, 4 IIB, 1 IIIB, 9 IIIC1, 3 IIIC2, 1 IVA, and 3 IVB) (Table 3; Table S7). The median time between initial HG‐CIN diagnosis and recurrent CIN2+ was 10.1 months (range: 3.0‐241.4 months). The 5‐year cumulative recurrent CIN2+ rate was 14.4% after conization in the non‐S group (which was not significantly different from that in the SN or SP groups, *P* = .319) (Figure 2).

**TABLE 3:** Distribution of FIGO stages of those progressed to invasive cervical cancer by surveillance group and non‐surveillance group

Variable	Surveillance group		Non‐surveillance group		*P* value^a^
	(n = 6)		(n = 57)		
	n	(%)	n	(%)	
FIGO stage					.882
IA1	4	(66.7)	15	(26.3)	
IA2	0	(0)	5	(8.8)	
IB1	2	(33.3)	10	(17.5)	
IB2	0	(0)	2	(3.5)	
IB3	0	(0)	1	(1.8)	
IIA1	0	(0)	3	(5.3)	
IIA2	0	(0)	0	(0)	
IIB	0	(0)	4	(7.0)	
IIIB	0	(0)	1	(1.8)	
IIIC1	0	(0)	9	(15.8)	
IIIC2	0	(0)	3	(5.3)	
IVA	0	(0)	1	(1.8)	
IVB	0	(0)	3	(5.3)	
FIGO stage					.033
1A‐IB1	6	(100)	30	(52.6)	
IB2‐IV	0	(0)	27	(47.4)	

^a^ Fisher's exact test.

**FIGURE 3:**
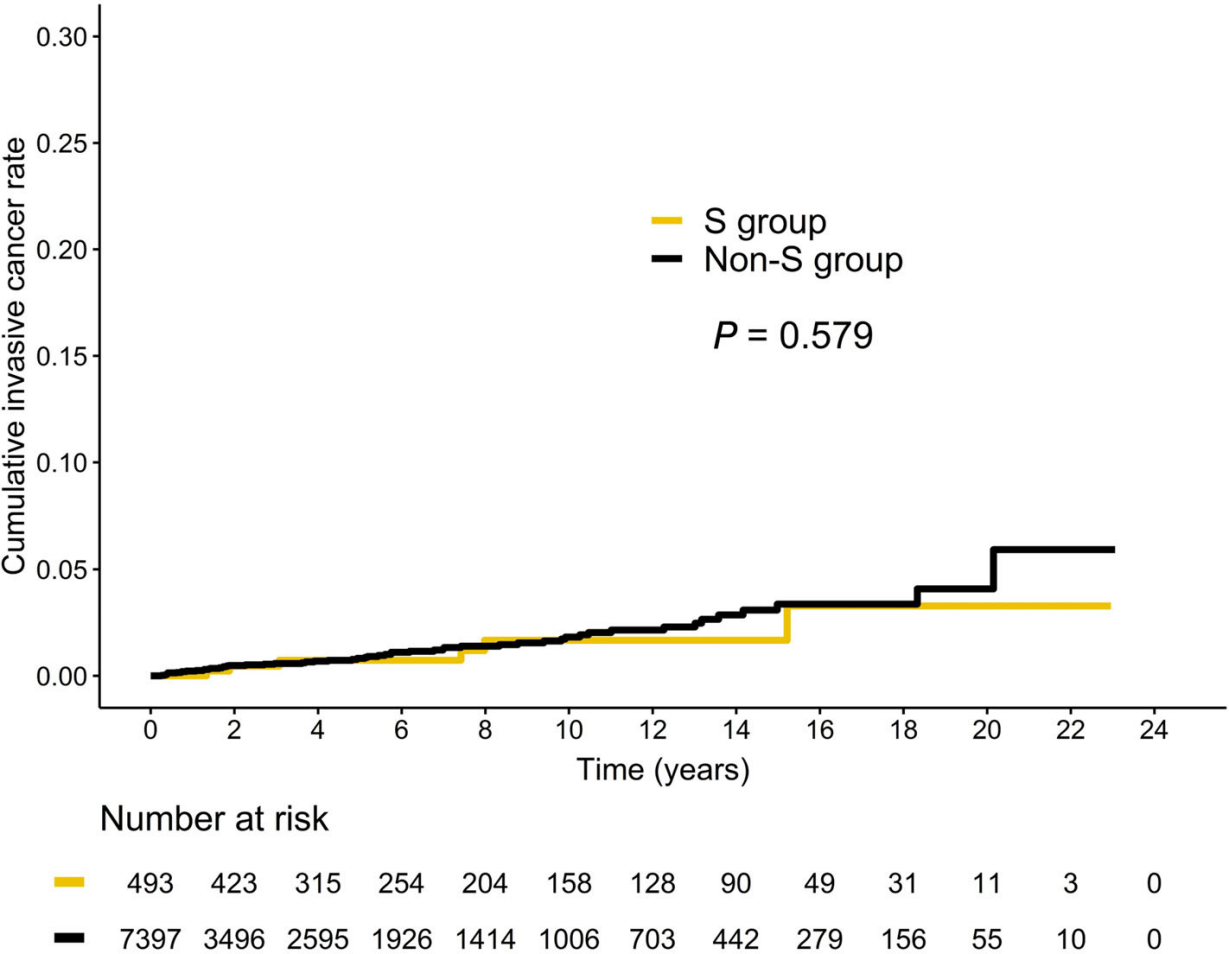
Kaplan‐Meier curves of cumulative invasive cervical cancer rates of surveillance (S) group vs non‐surveillance (non‐S) group

The median time to progression to invasive cervical cancer (S group: 62.9 months [range: 16.0‐182.6 months], non‐S group: 57.7 months [range: 3.0‐241.8 months]; *P* = .574) and the 5‐year cumulative rates (S group: 0.7%, non‐S group: 0.8%; *P* = .579) was not significantly different (Figure 3). However, the distribution of FIGO stages was significantly different (the non‐S group had more advanced stages than the S group; *P* = .033) (Table 3).

All the 84 S group patients with recurrence/progression had HPV genotyped in paired FFPE tissues. Of the 84 patients with paired HPV results, 47 (56.0%) developed concordant type‐specific persistent HPV infections, 37 (44.0%) had discrepant HPV types, and 19 (22.6%) had new HPV genotype(s) (7 [8.3%] of them had HPV that belonged to 9‐valent vaccine types). Among the 37 patients with discrepant results, 18 retained original types, although some patients acquired new types, or in some patients, some type(s) disappeared; therefore, type‐specific persistence was 77.4% (65/84) (Table S8). Among the 57 non‐S group patients who showed progression to cancer, 56 had paired HPV results. Among these 56 patients, 48 (85.7%) developed concordant type‐specific persistent HPV infections, 8 had discrepant results between HG‐CIN and cancerous tissue samples (4 retained the original type [persistent] in the cancerous tissue, and 4 developed a new vaccine type HPV); thus, type‐specific persistence was 92.9% (52/56) (Table S9).

The time‐dependent HPV infection status distribution of the S group patients is presented in Table S10. To sum up, the total clearance rate with or without interim events was 55.6%, the type‐specific persistence rate was 14.4%, and the new acquisition rate was 28.0% at the end of follow‐up. For patients with at least one post‐conization co‐test follow‐up visit (n = 425), the 5‐, 10‐, and 15‐year cumulative CIN2+ rates continued to rise to 43.2%, 64.8%, and 71.9%, respectively, as revealed by HPV+/cytology+ results at the first follow‐up visit. Additionally, recurrent CIN2+ rates were low among patients with ≥2 negative co‐tests (Table S11; Figure S6). However, some patients developed new HPV infections or original types re‐emerged after two or more negative co‐test results (Tables S12 and S13).

## 4 DISCUSSION

In our study, the 5‐year cumulative recurrent CIN2+ rate of 14.8% was obtained, which is generally consistent with the rates in our previous study^10^ and relevant literature.^15^, ^16^, ^17^ The risk of recurrence/progression did not plateau at 10 or 15 years in the non‐study cohort (Figure 2). Late recurrences of CIN2+ >5 years after conization were not uncommon (23.8%). Among patients with one negative co‐test after conization, the 15‐year cumulative CIN2+ rate was 17.3%. We used HPV whole genome PCR to examine patients who were found to be HPV‐negative by HPV Blot and E6 type‐specific PCR, yet 6.7% remained HPV‐negative. None of the patients with HPV‐negative HG‐CIN showed recurrence or progression. Nevertheless, new acquisition occurred in 28.0% of the patients at the end of follow‐up. Moreover, 77.4% (65/84) of the S group patients with recurrent CIN2+ had persistent type‐specific HPV infections. Although 19 (22.6%) of them had developed new HPV genotypes, only 7 were detected with HPV that belonged to 9‐valent vaccine types (ie, 8.3% [7 of 84] of CIN2+ cases might have been prevented if vaccination had been administered after conization).

In the literature, approximately 5% to 25% of patients developed residual or recurrent disease after conization for HG‐CIN.^15^, ^16^, ^17^, ^18^, ^19^ In our study, the incidence of recurrent/residual disease varied across different age groups and post‐treatment HPV statuses.^15^, ^16^, ^17^ In a Dutch nationwide registry, patients diagnosed with CIN3 had a long‐lasting increased risk of multiple HPV‐related anogenital tract precancers and cancers other than cervical and oropharyngeal cancer.^18^

The median value of HPV persistence tended to decline with increasing follow‐up time up to 24 months,^12^ while a Danish study discovered that in the first 5 years, the risk of CIN2+ in HPV‐negative patients at 3 to 4 months after conization for HG‐CIN was similar to that in HPV‐negative women in the routine screening population; however, 6 to 7 years later, higher risk was noted in the former group.^16^ Notably, none of these studies have included genotype information. Rebolj et al used a Dutch nationwide pathology registry data to examine the risk of cervical cancer in patients with CIN who returned to undergo routine screening after receiving post‐treatment consecutive normal Pap smear results. They reported that the adjusted HR (4.2, 95% CI 2.7‐6.5) of these patients remained significantly higher than that of the general population.^19^ In a study of 3273 patients aged 25+ years treated for HG‐CIN or adenocarcinoma in situ at Kaiser Permanente Northern California, the 5‐year recurrent CIN2+ rates were 2.4% among those with one negative post‐treatment co‐test and 1.5% among those with two negative co‐tests.^20^ In the United States, the guidelines of the American Society for Colposcopy and Cervical Pathology (ASCCP) recommend co‐testing at 12 and 24 months after treatment for HG‐CIN (BII). However, most of the non‐S patients or the SP group patients underwent follow‐up before enrollment through cytology alone because HPV testing is not reimbursed by the national insurance policy. ASCCP indicated that follow‐up is insufficient to determine post‐treatment outcomes or optimal long‐term follow‐up intervals for patients treated for HG‐CIN; therefore, future research is warranted.^21^ Although co‐test ≥2 times predicted a low 5‐year CIN2+ rate in our study, some patients developed new HPV infections or original types re‐emerged after two or more negative co‐test results.

Whether HPV vaccination can reduce the future risk of HPV‐related morbidities in patients treated for HG‐CIN has been controversial. However, a retrospective study demonstrated that only 2.5% of patients who received the quadrivalent HPV vaccine after LEEP developed recurrent CIN2/3 compared to 7.2% of those who did not receive the quadrivalent HPV vaccine after LEEP.^22^ Another non‐randomized prospective study demonstrated that the 4‐year risk of recurrent CIN2/3 was significantly reduced in the vaccine group compared to the non‐vaccine group (1.2% vs 6.4%, *P* = .0112).^23^ However, in the Costa Rica HPV Vaccine Trial, Hildesheim et al found no evidence that the vaccine increases the clearance of HPV infection or reduces the incidence of cytological/histological abnormalities associated with the HPV types present at enrollment. Additionally, they found that the vaccine did not reduce recurrence in patients treated for HG‐CIN.^24^ A Danish population‐based study also found that patients who received HPV vaccination 3 months before or 1 year after conization had a non‐significant HR (HR_adjusted_ = 0.86, 95% CI 0.67‐1.09) of recurrent CIN2+ compared to non‐vaccinated patients.^25^ In our study, the estimated reduction of future CIN2+ was estimated to be <10%.

In our study, we conducted a long‐term follow‐up of HPV genotype changes in relation to the recurrence and progression of HG‐CIN after conization. Our study had the following limitations: (a) The SP group might have had some advantage over the non‐S group because patients who showed progression to invasive cancer during follow‐up were not enrolled into the SP group; (b) many patients were lost to follow‐up in the non‐S group (median follow‐up: 19.8 months); and c) the cutoff of months to exclude non‐S cases with cancer diagnosis from the study was arbitrary; however, this may have inflated the percentage of the persistent HPV genotype among those patients with progression to cancer. Apparently, the cumulative CIN2+ rates were underestimated for both the S and non‐S groups. Another ongoing study using data from the Health and Welfare Data Science Center, Taiwan, will improve the recurrence/progression rate estimation. Additionally, the data on vaginal co‐tests were not analyzed in our study to avoid overwhelming readers and will be reported in a separate paper.

In conclusion, the recurrent CIN2+ rate was significantly higher among patients with HPV‐positive results than those with HPV‐negative results in the initial conization samples. Active surveillance might reduce the severity of those progresses to invasive cancer. Emergence of new oncogenic HPV infections is a significant threat; therefore, vaccination against the remaining hr‐HPV types could be included in the care provided to these patients. Because a majority of those with recurrent CIN2+ develop type‐specific persistent HPV infections, effective therapeutic vaccines remains an unmet medical need.

## CONFLICT OF INTEREST

The authors declared no potential conflicts of interest.

## ETHICS STATEMENT

The study was approved by the IRB, and all the S group patients gave their written consent.

## Supporting information


**Appendix** **S1**: Supporting Information

## Data Availability

The data will be made available upon reasonable request.
