# Supplementary material for: Role of human papillomavirus status after conization for high‐grade cervical intraepithelial neoplasia
Source: Int J Cancer. 2020 Sep 1;148(3):665–72. doi: 10.1002/ijc.33251 (PMC7754315; doi:10.1002/ijc.33251)
Supplement: Supplementary file 1 — Appendix S1: Supporting Information [file IJC-148-665-s001.pdf]

Role of human papillomavirus status after conization for high-grade cervical intraepithelial neoplasia

Huei-Jean Huang[1,2], Hsiu-Jung Tung[1,2], Lan-Yan Yang[3], Angel Chao[1,2], Yun-Hsin Tang[1,2], Hung-Hsueh Chou[1,2], Wei-Yang Chang[3], Ren-Chin Wu [4], Chu-Chun Huang[2], Chiao-Yun Lin [2], Min-Jie Liao[1,2], Wei-Chun Chen[1,2], Cheng-Tao Lin [1,2], Min-Yu Chen[1,2], Kuan-Gen Huang[1,2], Chin-Jung Wang[1], Ting-Chang Chang[1,2], Chyong-Huey Lai[1,2]

Contents

Supplementary methods 3

Supplementary references 4

Supplementary tables and figures 5

Supplementary Table 1. Primers for whole-genome amplification and sequencing of HPV16 5

Supplementary Figure 1. Schematic diagram of the HPV-16 PCR product from the Caski cell line 5

Supplementary Table 2. Primers for whole-genome amplification and sequencing of HPV18 6

Supplementary Figure 2. Schematic diagram of the HPV-18 PCR product from the HeLa-S3 cell line 6

Supplementary Table 3. Primers for whole-genome amplification and sequencing of HPV52 7

Supplementary Figure 3. Schematic diagram of the HPV-52 PCR product from a HPV-52 positive swab control specimen. 7

Supplementary Table 4. Primers for whole-genome amplification and sequencing of HPV58 8

Supplementary Figure 4. Schematic diagram of the HPV-58 PCR product from a HPV-58 positive swab control specimen. 8

Supplementary Table 5. Type-specific HPV recurrence/progression rates in surveillance group 9

Supplementary Figure 5. Kaplan-Meier curves of cumulative recurrence/progression by surveillance-new (SN) group, surveillance-previous (SP) group. 10

Supplementary Table 6. Clinical characteristics and HPV types in those progressed to cancer in Surveillance group (n=6) 11

Supplementary Table 7. Clinical characteristics and HPV types in those progressed

in non-Surveillance group (n = 57) 12
Supplementary Table 8. Discrepant HPV genotypes in S group who had a recurrence/progression (n=37) 15
Supplementary Table 9. Discrepant HPV genotypes in Non-S group who progressed to cancer (n=8) 19
Supplementary Table 10. The time-dependent HPV infection status of the surveillance group patients 20
Supplementary Table 11. 5-year, 10-year and 15-year cumulative recurrent CIN2+ rates according to cotest results after conization in the surveillance group (n = 425) 22
Supplementary Figure 6. Cumulative recurrent CIN2+ rates according to cotest results after conization. 24
Supplementary Table 12. HPV reemergence[a] or acquisition[b] after 2 or ≥3 consecutive co-test negative results in the SN group 25
Supplementary Table 13. HPV re-emergence[a] or acquisition[b] after 2 or ≥3 consecutive co-test negative results in the SP group 27

Supplementary methods

Cell line

HeLa S3 (RRID: CVCL_0058) and Ca Ski (RRID: CVCL_1100) cell lines were obtained from ATCC. The cell lines have recently (July 2020) been authenticated using short random repeat profiling by the Department of Laboratory Medicine, Chang Gung Memorial Hospital Linkou Medical Center (Taiwan).

The International Cell Line Authentication Committee has set guidelines for interpretation: cells with 80–100% allelic matching come from the same donor. (http://standards.atcc.org/kwspub/home/the_international_cell_line_authentication_committee-iclac_/Authentication_SOP.pdf)

According to Int J Cancer. 2010 Jan 1;126(1):303-4. (link: https://onlinelibrary.wiley.com/doi/full/10.1002/ijc.24999), HeLa-S3 and Caski with 94% allelic matching come from the same donor in the ATCC database.

HPV whole genome by direct sequencing

For HPV16 whole genomes were amplified by overlapping polymerase chain reaction (PCR), the PCR primers were designed by alignment of HPV16 genomes determined by modifying from Cullen et al.[1] HPV 16 primers were employed to amplify the entire 7905 bp HPV16 genome as 35 almost overlapping amplicons ranging in size from 160 bp to 295 bp **(Supplemental Table 1)**. DNA extraction from the Caski cervical cancer cell lines served as HPV16 positive control **(Supplementary Figure 1)**.

For HPV18 whole genomes were amplified by overlapping PCR, the PCR primers were designed by alignment of HPV18 genomes determined by using Primer 3 software. HPV 18 primers were employed to amplify the entire 7857 bp HPV18 genome as 36 almost overlapping amplicons ranging in size from 104 bp to 257 bp **(Supplemental Table 2)**. DNA extraction from the HeLa-S3 cervical cancer cell lines served as HPV18 positive control **(Supplementary Figure 2)**. Since HeLa-S3 cell lines are known to contain partial HPV genome due to deletions of 2.5kb region,[2] which was consistent with our method. Therefore, total 12 primers were excluded from further analysis.

For HPV52 whole genomes were amplified by overlapping PCR, the PCR primers were designed by alignment of HPV52 genomes determined by using

Primer 3 software. HPV 58 primers were employed to amplify the entire 7942 bp HPV52 genome as 25 almost overlapping amplicons ranging in size from 190 bp to 265 bp **(Supplemental Table 3)**. DNA extraction from the swab control specimen served as HPV52 positive control **(Supplementary Figure 3).**

For HPV58 whole genomes were amplified by overlapping PCR, the PCR primers were designed by alignment of HPV58 genomes determined by using Primer 3 software. HPV 58 primers were employed to amplify the entire 7824 bp HPV58 genome as 30 almost overlapping amplicons ranging in size from 200 bp to 450 bp **(Supplemental Table 4)**. DNA extraction from the swab control specimen served as HPV58 positive control **(Supplementary Figure 4)**.

The PCR conditions were as follows: initial denaturation and hot start at 95 ℃ for 10 minutes; 40 cycles at 95 ℃ for 30 seconds, at 50 ℃ for 40 seconds and at 72 ℃ for 30 seconds; final extension at 72 ℃ for 5 minutes.

The PCR products were obtained and used for directed sequencing.[3]

Supplementary references

1. Cullen M, Boland JF, Schiffman M, Zhang X, Wentzensen N, Yang Q, Chen Z, Yu K, Mitchell J, Roberson D, Bass S, Burdette L, et al. Deep sequencing of HPV16 genomes: A new high-throughput tool for exploring the carcinogenicity and natural history of HPV16 infection. Papillomavirus Res 2015;1:3-11.

2. Lagström S, Umu SU, Lepistö M, Ellonen P, Meisal R, Christiansen IK, Ambur OH, Rounge TB. TaME-seq: An efficient sequencing approach for characterisation of HPV genomic variability and chromosomal integration. Sci Rep 2019;9:524.

3. Chao FY, Chao A, Huang CC, Hsueh S, Yang JE, Huang HJ, Wang LC, Lin CT, Chou HH, Lai CH. Defining detection threshold and improving analytical proficiency of HPV testing in clinical specimens. Gynecol Oncol 2010;117:302-7.

Supplementary tables and figures

Supplementary Table 1. Primers for whole-genome amplification and sequencing of HPV16

Primer name	Primer sequence (5'-3')	Primer name	Primer [illegible]quence (5'-3')	PCR product (bp)
HPV16_1_F	ACAGTTACTGCGACGTGAGG	HPV16_1_R	TGGAATCTTTGCTTTTTGTCC	253
HPV16_2_F	TCAAAAGCCACTGTGTCCTG	HPV16_2_R	TTCATCCTCCTCCTCTGAGC	232
HPV16_3_F	TTGCAACCAGAGACAACTGA	HPV16_3_R	TTCTGAGAACAGATGGGGCAC	228
HPV16_4_F	GCGTACAAAGCACACACGTA	HPV16_4_R	CTGTCATTTTCGTTCTCGTCA	206
HPV16_5_F	ACGGGATGTAATGGATGGTT	HPV16_5_R	TGTTGTTTTGCTTCCTGTGC	180
HPV16_6_F	TTTAACACAGGCAGAAACAGAGAC	HPV16_6_R	CGCCCTTCTACCTGTAACATC	252
HPV16_7_F	GCTGCAAAAAGGAGATTATTTG	HPV16_7_R	ATTGCTGCCTTTGCATTACT	238
HPV16_8_F	TGCCAAACACCACTTACAAA	HPV16_8_R	CATTCCCCATGAACATGCTA	241
HPV16_9_F	GGTGTATTGCTGCATTTGGA	HPV16_9_R	TACGCAATTTTGGAGGCTCT	214
HPV16_10_F	ACACGCCAGAATGGATACAA	HPV16_10_R	CACATTGTTGCACAATCCTTT	203
HPV16_11_F	TGCACAATTGGCAGACACTA	HPV16_11_R	ACCTGTGTTAGCTGCACCAT	290
HPV16_12_F	AGATGTGATAGGGTAGATGATGGAG	HPV16_12_R	TTGTCATCTATGTAGTTCCAACAGG	295
HPV16_13_F	GCAGATGCCAAAATAGGTATG	HPV16_13_R	ACTGGATTTCCGTTTTCGTC	242
HPV16_14_F	GATTGGTGGTGTTTACATTTCC	HPV16_14_R	CATTCTAGGCGCATGTGTTT	244
HPV16_15_F	AAAACGATGGAGACTCTTTGC	HPV16_15_R	AGTTGCAGTTCAATTGCTTGT	215
HPV16_16_F	GTGCCAACACTGGCTGTATC	HPV16_16_R	TGCATATGTCTCCATCAAACTG	185
HPV16_17_F	AAACATGGATATACAGTGGAAGTGC	HPV16_17_R	ATTACCTGACCACCCGCATG	220
HPV16_18_F	TGCAGTTTAAAGATGATGCAGA	HPV16_18_R	CGCTGGATAGTCGTCTGTGT	192
HPV16_19_F	CCATAGTACATTTAAAAGGTGATGC	HPV16_19_R	CGCCAGTAATGTTGTGGATG	249
HPV16_20_F	GTTCTGCAAAACGCACAAA	HPV16_20_R	GGGGTCTTACAGGAGCAAGT	242
HPV16_21_F	GTACAGGCGGACGCACTG	HPV16_21_R	GGGATTATTATGTGTAGTAACAGTAGT	272
HPV16_22_F	TCAACTGATACCACACCTGCT	HPV16_22_R	GAGACCCTGGTATGGGTGTG	226
HPV16_23_F	GACCCTGCTTTTGTAACCACTC	HPV16_23_R	GTACGCCTAGAGGTTAATGCTGG	160
HPV16_24_F	CATGTTACGAAAACGACGTAAA	HPV16_24_R	CCAAACTTATTGGGGTCAGG	273
HPV16_25_F	CTTGCAGTTGGACATCCCTA	HPV16_25_R	CACACCTAATGGCTGACCAC	199
HPV16_26_F	GTTTGGGCCTGTGTAGGTG	HPV16_26_R	TTCCCCTATAGGTGGTTTGC	188
HPV16_27_F	AGCAAATGCAGGTGTGGATA	HPV16_27_R	TCCAGTGGAACTTCACTTTTG	241
HPV16_28_F	TGGTGAAAATGTACCAGACGA	HPV16_28_R	GATATGGCAGCACATAATGACA	225
HPV16_29_F	ATGGCATTTGTTGGGGTAAC	HPV16_29_R	ACCAAAATTCCAGTCCTCCA	234
HPV16_30_F	TGTGCAAAATAACCTTAACTGC	HPV16_30_R	TGCGTCCTAAAGGAAACTGA	245
HPV16_31_F	GGAGGCACACTAGAAGATACTTATAGG	HPV16_31_R	GAGGTGGTGGGTGTAGCTTTTC	218
HPV16_32_F	AAGGCCAAACCAAAATTTACA	HPV16_32_R	GCATGACACAATAGTTACACAAGC	233
HPV16_33_F	GTTTGTATGTGCTTGTATGTGCTTG	HPV16_33_R	CGGTTGAAGCTACAAAATGGC	233
HPV16_34_F	GCCATTTTGTAGCTTCAACCG	HPV16_34_R	CAAGCCAAAAATATGTGCCTAAC	264
HPV16_35_F	CAAACCGTTTTGGGTTACAC	HPV16_35_R	ATGCATAAATCCCGAAAAGC	288

HPV16 complete genome sequence is based on the NCBI Reference Sequence: NC_001526.2
(HPV16 http://www.ncbi.nlm.nih.gov/nuccore/NC_001526.2)

Supplementary Figure 1. Schematic diagram of the HPV-16 PCR product from the Caski cell line

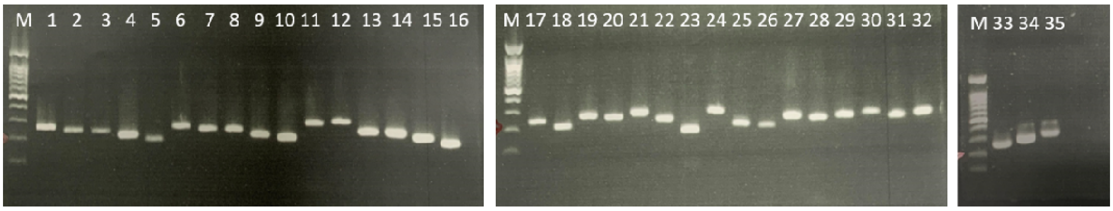

Supplementary Table 2. Primers for whole-genome amplification and sequencing of HPV18

Primer name	Primer sequence (5'-3')	Primer name	Primer sequence (5'-3')	PCR product (bp)
HPV18_1F	CCTGGGCAATATGATGCTACCAA	HPV18_1R	GAACACCAAAGTTCCAATCCTCTAA	165
HPV18_2F	CACTTCACTGCAAGACATAG	HPV18_2R	CACCGCAGGCACCTTATTA	257
HPV18_3F	CCAGAAACCGTTGAATCCAG	HPV18_3R	GAAGGTCAACCGGAATTTCA	237
HPV18_4F	TGAAATTCCGGTTGACCTTC	HPV18_4R	GGATGCACACCACGGACACAC	252
HPV18_5F	CAATGGCTGATCCAGAAGGT	HPV18_5R	TGTGCATCATTGTGGACCTC	240
HPV18_6F	GCAGCACAGAAAACAGTCCA	HPV18_6R	CGTACTGCCGCCACTACATA	220
HPV18_7F	TATAGACAACGGGGGCACAG	HPV18_7R	CCCAATCTGTACACGTGGTTT	229
HPV18_8F	CACATATGGGCTATCATTTACAGA	HPV18_8R	TTGTAACGCAACAGGGCTAA	206
HPV18_9F	AACCACCAAAATTGCGAAGT	HPV18_9R	CATTGCTGTTGCTGTCTGCT	242
HPV18_10F	AGCAGACAGCAACAGCAATG	HPV18_10R	TGTTGGTATCGCAGGAATTG	203
HPV18_11F	CAATTCCTGCGATACCAACA	HPV18_11R	CGGTTCCAACCAAAAATGAC	206
HPV18_12F	CATTTTTGGTTGGAACCGTTA	HPV18_12R	TGGCCATCTATTATCCTTTGC	212
HPV18_13F	TCCAGCAAAGGATAATAGATGG	HPV18_13R	AAGGGTTTCCTTCGGTGTCT	199
HPV18_14F	GAAGGAAACCCTTTCGGAAC	HPV18_14R	CTTGTAGGGCCATTTGCAGT	241
HPV18_15F	ACTGCAAATGGCCCTACAAG	HPV18_15R	GTAGCCGTTTTGTCCCATGT	230
HPV18_16F	ACATGGGACAAAACGGCTAC	HPV18_16R	GAGTAGCGGATACCGTGTCG	201
HPV18_17F	CTGCTACACGACCTGGACAC	HPV18_17R	GGTCGCTATGTTTTCGCAAT	200
HPV18_18F	TATCATCCACCTGGCATTGG	HPV18_18R	GGACATGGCAGCACACATAC	233
HPV18_19F	GTGCGTATGCATGGGTATTG	HPV18_19R	GCACGGTGGGATACCATACT	239
HPV18_20F	ACGGGCTTCGGTAACTGACT	HPV18_20R	TGTAGGACCAACATCCACCA	231
HPV18_21F	TCCCCCAGTGGTTATTGAAC	HPV18_21R	CGGATCAGAAAATGCAGGAT	231
HPV18_22F	GCATTTTCTGATCCGTCCAT	HPV18_22R	TACTGTAAAGGCGGGGACCT	204
HPV18_23F	GGTCCCCGCCTTTACAGTAG	HPV18_23R	ACGCCTGGATGTTAAAGCAG	206
HPV18_24F	TCCGTCTACATAGGCCTGCT	HPV18_24R	ACTGCAGGGTCCATGTCATC	237
HPV18_25F	CACGGAGGACAATGACTTGTT	HPV18_25R	ACAATGGGCCATACAGAGGT	227
HPV18_26F	GCCCCTGCCTCTACACAGTA	HPV18_26R	GAGCTGCCAGCATGATAAAA	227
HPV18_27F	TTATGTGACTCGCACAAGCA	HPV18_27R	AATTTCCACTCCAGCACAGG	243
HPV18_28F	CGTGGTCAGCCTTTAGGTGT	HPV18_28R	GGACGCGATTTACAAGCAGT	211
HPV18_29F	CCTTTATCACAGGGCGATTG	HPV18_29R	GCCTAGCAAAAAGCTGCTCA	240
HPV18_30F	GCAGCTTTTTGCTAGGCATT	HPV18_30R	GCCAGCAAACACCATTGTTA	217
HPV18_31F	CTGTCAAAAGGATGCTGCAC	HPV18_31R	ACGCTTGGCAGGTTTAGAAG	216
HPV18_32F	TCTAAACCTGCCAAGCGTGT	HPV18_32R	TTACTCACTAGGGCGCAACC	236
HPV18_33F	TGCGCCCTAGTGAGTAACAA	HPV18_33R	TTTGCAATAGTGCCAGCGTA	213
HPV18_34F	AGGCGCACCTGGTATTAGTC	HPV18_34R	CCAACCTATTTCGGTTGCAT	242
HPV18_35F	CACTTCACTGCAAGACATAG	HPV18_35R	CACCGCAGGCACCTTATTA	213
HPV18_36F	AAGAAAACGATGAAATAGATGGA	HPV18_36R	GGCTTCACACTTACAACACA	104

HPV18 complete genome sequence is based on the NCBI Reference Sequence: AY262282.1
(HPV18 http://www.ncbi.nlm.nih.gov/nuccore/AY262282.1)

Supplementary Figure 2. Schematic diagram of the HPV-18 PCR product from the HeLa-S3 cell line

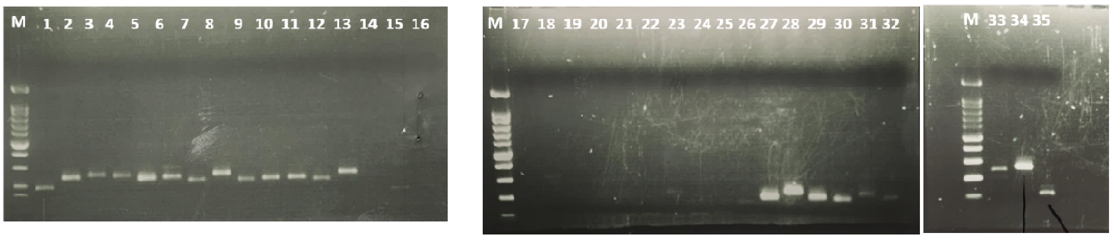

Supplementary Table 3. Primers for whole-genome amplification and sequencing of HPV52

Primer name	Primer sequence(5'-3")	Primer name	Primer sequence(5'-3")	PCR product (bp)
HPV52-1F	GGGATGTACAGGCTGGTTTG	HPV52-1R	AATCATCCTCCCCTTCCTGT	200
HPV52-2F	TACAAGCAGTCCGGAAAGTG	HPV52-2R	TACTTTGCCAGTCGCCATTT	203
HPV52-3F	TGTGACAGAGGCGTGCTTAT	HPV52-3R	TCTATCCATTCTGGGGTGGT	218
HPV52-4F	AAATGGTGCAATGGGCATA	HPV52-4R	CCTCCAATCTCCACCATCAT	245
HPV52-5F	TATGGACCTGCAAACACAGG	HPV52-5R	TCCATCCAGTGCATTTCTCA	201
HPV52-6F	TGCAGGAACAGATCCTAGGTG	HPV52-6R	CGGTATCGACTCCATCGTTT	200
HPV52-7F	CGCACAAATTGAACATTGGA	HPV52-7R	TTCTGCACGCCACATTTCTA	235
HPV52-8F	CATGGGTATACAATAACAGTGCAA	HPV52-8R	CAATTACCTGACCACCCACA	244
HPV52-9F	GTCCACCTATGCACCGAAAC	HPV52-9R	GTATGTGCAACCCGTCCTTT	221
HPV52-10F	TGGCATTGGACCAGTAATGA	HPV52-10R	TGGCTTGTGTTGGGTGTTTA	202
HPV52-11F	AACGTGCTTCTGCAACACAG	HPV52-11R	TAGTGGGAGGACGAGTGGAC	213
HPV52-12F	CCCATTGGTCCCTTAGAACC	HPV52-12R	GACCAGATGCTTCTGCAGGT	253
HPV52-13F	CTGACAGCAGCAGTGTAACAAG	HPV52-13R	CCGGTGCAGGTAAAAGTTGT	219
HPV52-14F	GCCTGCATTAACCTCTCGAA	HPV52-14R	TTGCTGCAAAGAATCTGCAT	241
HPV52-15F	CCACGTTTCACTTACCTTCCA	HPV52-15R	GTTTACGCCTGCGACGTAGT	252
HPV52-16F	CCACTGTGTACCTGCCTCCT	HPV52-16R	GGGTCCGGCAATTTAATTCT	223
HPV52-17F	ATTGGTAGGGGACAGCCTTT	HPV52-17R	GGTTCCCTTACCCCAATGTT	201
HPV52-18F	GAAATCCTGGGGATTGTCCT	HPV52-18R	TGTCTAACAAACATTTGCTCACG	238
HPV52-19F	CCCTGTGCCAGGTGATTTAT	HPV52-19R	TGCTACGAGTGGTATCCACAA	218
HPV52-20-F	ATGTTTTGGGAGGTGGATTT	HPV52-20R	CATGACACAGACAATTACCCAAC	200
HPV52-21F	TGTGTGTGTACTGTGTTGTTTGC	HPV52-21R	AGGGCAGGAGATGCAATAGA	201
HPV52-22-F	TGTATGTGTGTGCATGTTTGTTG	HPV52-22R	CAAAATGGTACAAAATGGAGAGT	254
HPV52-23F	TGTCCCGCCTAAACTGACTT	HPV52-23R	AACCGTAACCGGTCGTGTAG	233
HPV52-24F	ATGTTTGAGGATCCAGCAACA	HPV52-24R	TACACACGCCATATGGATTAT	190
HPV52-25F	GCGTGTGTATTATGTGCCTACG	HPV52-25R	TTACACTTGGGTCACAGGTC	265

HPV52 complete genome sequence is based on the NCBI Reference Sequence: NC_001592.1
(HPV 52 http://www.ncbi.nlm.nih.gov/nuccore/NC_001592.1?report=genbank)

Supplementary Figure 3. Schematic diagram of the HPV-52 PCR product from a HPV-52 positive swab control specimen.

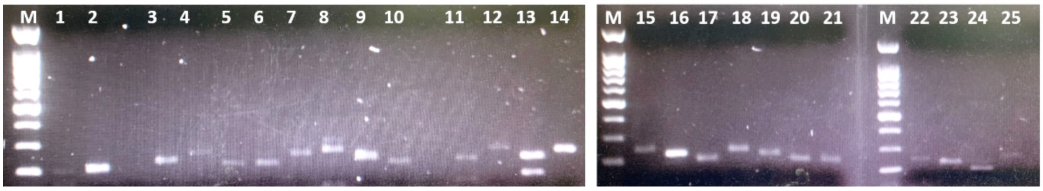

Supplementary Table 4. Primers for whole-genome amplification and sequencing of HPV58

Primer name	Primer sequence (5'-3')	Primer name	Primer sequence (5'-3'_	PCR product (bp)
HPV58-1F	GTGTAACCGAAAACGGTCTGA	HPV58-1R	ACCTCAGATCGCTGCAAAGT	200
HPV58-2F	GACCATTGTGTCCACAAGAAAA	HPV58-2R	CATCCTCGTCTGAGCTGTCA	248
HPV58-3F	AAGGTACAAACGGGGTAGGG	HPV58-3R	TCGTTTTAGTGCACACACAGC	248
HPV58-4F	GGGGTGGACGATATAAATGC	HPV58-4R	TGTGCCATCTGCTCAGTTTC	212
HPV58-5F	GTGGGGGCTAGTTCAGATGT	HPV58-5R	GGAGGGACTTATTCCATACCC	228
HPV58-6F	AAGTCCCTCCGTAGCAGAAA	HPV58-6R	CGTAATTTTGGTGGCTCGAT	210
HPV58-7F	GCAAGGGACAACACCAGAAT	HPV58-7R	TTTTGCTTGTGCATTGCTTC	205
HPV58-8F	TGGCGTTATGTGCAGACATT	HPV58-8R	GCTGGGCCACACAGTAACAT	222
HPV58-9F	TTTGGTTGCAGCCATTATCA	HPV58-9R	TGAATCTTTGCCTGCATTTG	200
HPV58-10F	CAAATGCAGGCAAAGATTCA	HPV58-10R	TCCTGCACTGCACTTAAACG	227
HPV58-11F	CGTTTAAGTGCAGTGCAGGA	HPV58-11R	TCTAATGCCATTTGCAGTTCA	221
HPV58-12F	GATGAATGGACATTGCAACAA	HPV58-12R	CCCCACATAGTCAACTTCTCC	201
HPV58-13F	TACATGGCAATGAAAAGACG	HPV58-13R	CGCTTTGTCCCCTGTGTACT	205
HPV58-14F	CCACGAGGAGGAGGACTACA	HPV58-14R	GTCGTTGTGTTTCCGTTGTG	247
HPV58-15F	CCACCCACTGTGCAAATAAG	HPV58-15R	CAAAGCAGCAACACCAACAC	226
HPV58-16F	GGGTCGGCTCTACGAATTTT	HPV58-16R	TTGTAGACCGTTTGTGTCTCAT	242
HPV58-17F	GCGTGCATCTGCTACACAAC	HPV58-17R	CCTCAGACGGTGGGGTACTA	215
HPV58-18F	TCCCCCAGTTACCGTTGATA	HPV58-18R	CGGATGGCTCAGTAAAGGAG	236
HPV58-19F	CCTCCTTTACTGAGCCATCC	HPV58-19R	ACTTGTTGGGTGTTGCGACT	223
HPV58-20F	AGTCGCAACACCCAACAAGT	HPV58-20R	GCGAGAGGTTAATGCAGGTC	201
HPV58-21F	CACTTCGTACTCGCAGTGGA	HPV58-21R	TGGCAAAGGACGTATGTGAG	237
HPV58-22F	TGGAACCTGGTCCAGACATT	HPV58-22R	AGGAGGCAGGTACACAGTGG	219
HPV58-23F	ACTTTTGGCTGTTGGCAATC	HPV58-23R	CAATGGCTGTCCCCTACCTA	220
HPV58-24F	GCACAGCCAGGGTCTGATA	HPV58-24R	AAAGTCCATGCATCCAAACC	219
HPV58-25F	TGGATGCATGGACTTTGGTA	HPV58-25R	ATAAAGGTCATCCGGGACAG	214
HPV58-26F	AATAGGGCTGGAAAACTTGG	HPV58-26R	AATAACTGATTGCCCCAGCA	206
HPV58-27F	ATGTTCCAGGACGCAGAG	HPV58-27R	TTACACTTGTGTTTGTCTACGTC	450
HPV58-28F	ATGAGAGGAAACAACCCAACG	HPV58-28R	TTATTGCTGTGCACAGCTAGG	297
HPV58-29F	ATGCACTGAAGTAACTAAGGAA	HPV58-29F	GCCTGGGAGGTAACAAATCTAT	240
HPV58-30F	ATGGAAATCCATTTGCAGTATG[illegible]	HPV58-30F	CTTTTGTTTAAATCCACATGCCT	204

HPV58 complete genome sequence is based on the NCBI Reference Sequence: D90400.1

(HPV58 http://www.ncbi.nlm.nih.gov/nuccore/D90400.1)

Supplementary Figure 4. Schematic diagram of the HPV-58 PCR product from a HPV-58 positive swab control specimen.

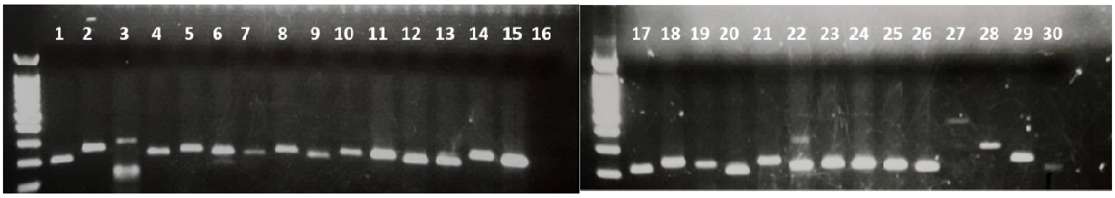

Supplementary Table 5. Type-specific HPV recurrence/progression rates in surveillance group

	HPV at initial conization	Recurrent CIN2+	Progression to invasive cancer
	n (%)	n (%)	n (%)
Total	493 (100)	84 (17.0)	6 (1.2)
HPV-	28 (5.7)	0 (0)	0 (0)
HPV+	465 (94.3)	84 (18.1)	6 (1.3)
Single	368 (74.6)	62 (16.8)	5 (1.4)
Multiple	97 (19.7)	22 (22.7)	1 (1.0)
HPV16 [a]	156 (31.6)	36 (23.1)	4 (2.6)
HPV18 [a]	27 (5.5)	4 (14.8)	1 (3.7)
HPV31 [a]	28 (5.7)	9 (32.1)	0 (0)
HPV33 [a]	32 (6.5)	9 (28.1)	0 (0)
HPV35 [a]	6 (1.2)	1 (16.7)	0 (0)
HPV39 [a]	13 (2.6)	1 (7.7)	0 (0)
HPV45 [a]	5 (1.0)	0 (0)	0 (0)
HPV51 [a]	25 (5.1)	3 (12.0)	0 (0)
HPV52 [a]	127 (25.8)	19 (15.0)	1 (0.8)
HPV56 [a]	12 (2.4)	4 (33.3)	0 (0)
HPV58 [a]	77 (15.6)	12 (15.6)	1 (1.3)
HPV59 [a]	3 (0.6)	0 (0)	0 (0)
HPV68 [a]	6 (1.2)	2 (33.3)	0 (0)
HPV82 [a]	7 (1.4)	0 (0)	0 (0)
Probable hr-HPVs[b]	18 (3.7)	3 (16.7) *	0 (0) **
Lr-HPVs [c]	39 (7.9)	9 (23.1) *	0 (0) **

HPV, human papillomavirus.

[a] Same woman can be counted more than once because of multiple infections.

[b] Probable hr-HPVs were HPV 26, 53, and 66.[1]

[c] Low-risk types are (6, 11, 32, 37, 42, 43, 44 , 54, 55, 61, 62, 67, 69, 70, 71, 72, 74, 81, 83, 84, L1AE5). [4,8,10,13]

* The rates of recurrent CIN2+ of probable hr-HPVs and lr-HPVs are not different (*P* = .967).

**Besides, none of them have cases progressed to cancer. We therefore grouped probable hr-HPVs with lr-HPVs in Table 2.

Supplementary Figure 5. Kaplan-Meier curves of cumulative recurrence/progression by surveillance-new (SN) group, surveillance-previous (SP) group.

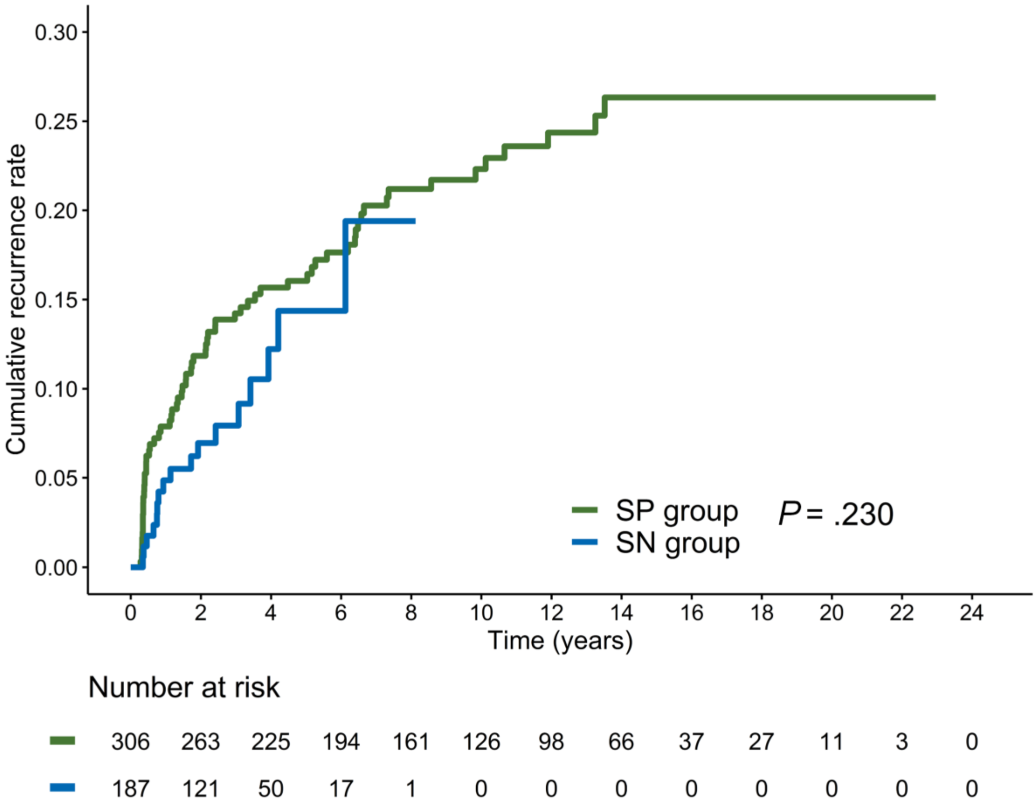

Supplementary Table 6. Clinical characteristics and HPV types in those progressed to cancer in Surveillance group (n=6)

Patient	Group	DX at first conization	Margin status	Endocervical curettage at conization	HPV type in HG-CIN	HPV type in cervical cancer	FIGO stage	Time from HG-CIN to invasive cancer (months)	HPV Infection status
C0058	SP	CIN3	-	Negative	18[a]	18	IA1	89.0	3-2
C0102	SP	CIN3	+	Negative	16	16	IB1	95.8	3-1
C0235	SP	CIN3	+	Negative	52	16	IA1	182.6	4-1
C0308	SN	CIN2	-	Negative	16	16	IA1	16.0	3-1
C0326	SN	CIN3	+	Negative	16	16	IB1	36.9	3-1
NV200	SN	CIN3	+	Negative	16,58	16,58	IA1	22.6	3-1

FIGO, International Federation of Gynecology and Obstetrics; *HPV*, human papillomavirus.

[a] This patient had HPV-18 CIN3 at 7 years before her enrollment (see also on Supplementary Table 11) who acquired HPV84 but subsequently cleared and had a recurrent CIN 3 at 7 months before enrollment and subsequent follow-up Pap smear was HSIL a hysterectomy was advised but declined by the patient. She was enrolled and HPV18+ with Pap SCC/biopsy CIN3 and treated with hysterectomy and final pathology revealed FIGO stage squamous cell carcinoma IA1.

Supplementary Table 7. Clinical characteristics and HPV types in those progressed in non-Surveillance group (n = 57)

Patient	DX	HPV type in HG-CIN	HPV type in cervical cancer	FIGO stage	Time from HG-CIN to invasive cancer (months)
32	CIN3	18	18	IA1	7.3
54	CIN2	31	negative	IIIC1	117.8
430	CIN3	16	16	IB1	15.0
659	CIN3	18	18	IA1	22.3
1026	CIN3	45	45	IIB	105.3
1032	CIN2	58	58	IA1	84.2
1145	CIN3	16	16	IA2	3.7
1922	CIN2	16	16	IIIC1	61.4
2108	CIN3	16	16	IA1	4.6
2168	CIN3	58	58	IIIB	158.1
2647	CIN2	16	16	IA1	57.7
2679	CIN3	16	16	IA1	27.1
2709	CIN3	16	16	IIIC1	83.8
3016	CIN3	18	18	IIIC1	132.1
3144	CIN3	16,52	16,18,52,58	IIIC1	35.4
3446	CIN3	18	18	IA1	21.7
4032	CIN2	16	16	IA1	8.8
4088	CIN3	58	58	IA1	4.8
4215	CIN3	16	16	IA2	17.0
4603	CIN3	16	16	IIA1	50.9
4672	CIN3	33	33	IA2	16.6
4704	CIN2	31	31	IIIC1	20.8
4729	CIN3	16	16	IA2	19.6

Patient	DX	HPV type in HG-CIN	HPV type in cervical cancer	FIGO stage	Time from HG-CIN to invasive cancer (months)
5259	CIN2	16	16	IB2	170.0
5552	CIN2	18	18	IB1	69.0
5594	CIN3	16	16	IA2	74.3
5595	CIN3	16,18	16,18	IA1	4.4
5740	CIN3	16	16	IIIC2	147.2
5748	CIN3	18	18	IB1	3.0
5812	CIN3	52	52	IA1	20.6
5871	CIN3	16	16	IA1	15.2
5997	CIN2	52	52	IA1	4.8
6311	CIN3	16	16	IIIC1	162.9
6339	CIN2	negative	16	IIIC1	119.0
6359	CIN3	58	58	IVB	123.1
6467	CIN3	45	45	IB1	69.0
6555	CIN3	70	70	IB1	46.9
6646	CIN3	58	58	IIIC2	100.0
6809	CIN3	16	NA*	IIIC1	179.8
7040	CIN3	58	58	IIB	44.4
7217	CIN2	18	18,33	IVB	65.2
7245	CIN3	59	59	IVB	219.9
7294	CIN2	58	58	IIA1	59.2
7515	CIN3	56	56	IB1	8.8
7525	CIN3	58	58	IB1	62.2
7631	CIN2	39	39	IB1	67.2
8067	CIN3	16	16	IB3	32.5

Patient	DX	HPV type in HG-CIN	HPV type in cervical cancer	FIGO stage	Time from HG-CIN to invasive cancer (months)
8110	CIN3	33	33	IB1	89.0
8119	CIN3	16	negative	IIB	10.2
8493	CIN2	33,81	33	IB1	12.9
8837	CIN3	33	33	IIIC2	112.8
9088	CIN2	16	16	IB2	4.8
9119	CIN2	33	33	IIB	156.2
9413	CIN3	16	16	IA1	241.8
9481	CIN2	52	52	IVA	125.6
9504	CIN3	16,66	16	IIA1	81.2
9587	CIN2	negative	45	IA1	43.3

*The tissue block of invasive cancer of this patient was missing.

Supplementary Table 8. Discrepant HPV genotypes in S group who had a recurrence/progression (n=37)

Patient	group	Dx	Margin status	Endocervical curettage at conization	HPV type in HG-CIN	HPV type in recur	Time to Recur (months)	Discrepant
C0156	SP	CIN3	NA	-	16,52	16	3.8	HPV16 persistent
C0155	SP	CIN3	NA	+	16,44	16	3.9	HPV16 persistent
NV131	SP	CIN3	+	-	16,52	16	6.2	HPV16 persistent
NV061	SP	CIN3	+	-	16,18,52	16	10.2	HPV16 persistent
C0220	SP	CIN3	no op record	no op record	16,31	16	53.7	HPV16 persistent
C0208	SP	CIN3	+	-	16	16,39[a]	13.3	HPV16 persistent , HPV39 new non-vaccine type
C0341	SP	CIN3	NA	+	16	16,42[a]	37.6	HPV16 persistent, HPV42, new non-vaccine type
NV413	SN	CIN3	+	+	16,39	16,33[a,b],39	7.8	HPV16,39 persistent, HPV33 new vaccine type
C0081	SP	CIN2	NA	-	18,58	18	20.6	HPV18 persistent
C0194	SP	CIN2	+	-	33,42	33	4.8	HPV33

Patient	group	Dx	Margin status	Endocervical curettage at conization	HPV type in HG-CIN	HPV type in recur	Time to Recur (months)	Discrepant
								persistent
C0189	SP	CIN3	no op record	no op record	33	33,56[a]	63.1	HPV33 persistent, HPV56, new non-vaccine type
C0001	SP	CIN2	NA	-	52	52,72 [a]	4.7	HPV52 persistent, HPV72, new non-vaccine type
C0292	SP	CIN2	no op record	no op record	52	16[b],52	16.1	HPV52 persistent, HPV16 new vaccine type
C0147	SP	CIN2	+	+	52,53,56	52,56	18.8	HPV52,56 persistent
C0270	SP	CIN3	-	-	51,56,68,81	56,81	8	HPV56,81 persistent,
NV046	SN	CIN3	+	+	58	51 [a],58	29	HPV58 persistent, HPV51 new non-vaccine type
C0009	SP	CIN2	-	+	18,58	58,62 [a]	4.2	HPV58 persistent, HPV62 new non-vaccine

Patient	group	Dx	Margin status	Endocervical curettage at conization	HPV type in HG-CIN	HPV type in recur	Time to Recur (months)	Discrepant
								type
C0165	SP	CIN2	NA	-	52,53,58	58,66 [a]	21.5	HPV58 persistent, HPV66 new non-vaccine type
C0174	SP	CIN2	+	+	16,42	negative	3.3	NA
C0132	SP	CIN3	+	-	52	negative	9.6	NA
C0157	SP	CIN2	no op record	no op record	52	negative	28.9	NA
C0206	SP	CIN2	no op record	no op record	58	negative	42.5	NA
C0279	SP	CIN3	+	+	52	negative	77.8	NA
C0026	SP	CIN2	NA	-	6,16	negative	88.2	NA
C0034	SP	CIN3	+	-	58	negative	102.8	NA
C0298	SP	CIN2	no op record	no op record	52	negative	121.5	NA
C0178	SP	CIN2	NA	-	56,58	negative	162.2	NA
C0035	SP	CIN3	+	-	33,58	51[a]	14.1	HPV51 new non-vaccine type
C0072	SP	CIN2	NA	-	16	56[a]	28.9	HPV56 new non-vaccine type
NV145	SN	CIN3	no op record	no op record	42,51,52	44 [a]	47.1	HPV44 new non-vaccine type
C0143	SP	CIN2	NA	-	31	53[a]	74.3	HPV53 new

Patient	group	Dx	Margin status	Endocervical curettage at conization	HPV type in HG-CIN	HPV type in recur	Time to Recur (months)	Discrepant
								non-vaccine type
NV016	SP	CIN2	no op record	no op record	16,58	51[a]	78.9	HPV51 new non-vaccine type
C0054	SP	CIN2	NA	+	26	16[a, b]	15.7	HPV16 new vaccine type
C0313	SP	CIN2	-	-	69	18[a, b]	17.4	HPV18 new vaccine type
C0061	SP	CIN2	NA	-	16	52[a, b]	40.1	HPV52 new vaccine type
C0209	SP	CIN2	+	no op record	33,56	58[a, b]	127.9	HPV58 new vaccine type
C0235	SP	CIN3	+	-	52	16[a, b]	159	HPV16 new vaccine type

[a]Nineteen (22.6%) of the 85 with recurrent CIN2+ harbored new HPV.

[b]Seven of these 19 belonged to 9-valent vaccine types.

Supplementary Table 9. Discrepant HPV genotypes in Non-S group who progressed to cancer (n=8)

Patient	DX	HPV type in HG-CIN	HPV type in cervical cancer	FIGO stage	Time from HG-CIN to invasive cancer (months)	Discrepant
9504	CIN3	16,66[b]	16	IIA1	81.2	HPV16 persistent
3144	CIN3	16,52	16,18[a],52[a],58[a]	IIIC1	35.4	HPV16 persistent, HPV18,52,58 new vaccine types
7217	CIN2	18	18,33[a]	IVB	65.2	HPV18 persistent, HPV33 new vaccine type
8493	CIN2	33,81[b]	33	IB1	12.9	HPV33 persistent,
8119	CIN3	16[b]	negative	IIB	10.2	NA
54	CIN2	31[b]	negative	IIIC1	117.8	NA
9587	CIN2	negative	45[a]	IA1	43.3	HPV45 new vaccine type
6339	CIN2	negative	16[a]	IIIC1	119.0	HPV16 new vaccine type

[a]Five harbored new HPVs.

[b]Four retained original type though acquired new types or some types disappeared.

Supplementary Table 10. The time-dependent HPV infection status of the surveillance group patients

Follow-up HPV status	SN group n (%)	SP group n (%)	S group n (%)
(1) Total clearance (+,-,-)	55 (29.4)	104 (34.0)	159 (32.3)
(2) Subsequent total clearance [a] (+,+,-) or (+,-,+,-)	32 (17.1)	83 (27.1)	115 (23.3)
(3)-1 Persistent with the original type(s) without acquisition of new type(s) (+,+,+) or (+,+)[b]	25 (13.4)	20 (6.5)	45 (9.1)
(3)-2 Persistent with the original type(s) with acquisition of new type(s) (+,++,++[c]) or (+,+,++[c])	11 (5.9)	15 (4.9)	26 (5.3)
(4)-1 Immediate clearance of the original type(s) but acquisition of new type(s) (+,-,+) or (+,-,++[c])	27 (14.4)	48 (15.7)	75 (15.2)
(4)-2 Subsequent clearance of the original type(s) but acquisition of new type(s) (+,+,-,+) or (+,+,-,++[c])	10 (5.3)	19 (6.2)	29 (5.9)
(5)-1 Initial HPV-negative of conization specimens but turn HPV-positive and persist at the end of follow-up (-,+,+) or (-,+,-,+)	0 (0)	2 (0.7)	2 (0.4)
(5)-2 Initial HPV-negative of conization specimens but turn HPV-positive but subsequent cleared during follow-up (-,+,-)	1 (0.5)	5 (1.6)	6 (1.2)
(6) Initial HPV-negative and remain HPV-negative during follow-up (-,-,-)	4 (2.1)	10 (3.3)	14 (2.8)
Unevaluable [d]	22 (11.8)	0 (0)	22 (4.5)

HPV, human papillomavirus; *SN*, surveillance new; *SP*, surveillance previous.

[a] For those who ultimately cleared despite earlier transient persistent or interval acquisition of HPV infections, they are designated (2) Subsequent total clearance.

[b] Those who had only one follow-up visit where an event of CIN2+ was noted.

[c] "++" indicate multiple types including original and newly acquired types. "+" is used for new acquisition of multiple types or single type without persistent of original type(s).

[d] Unevaluable: Those who have ≤1 follow-up visits unless they were HPV+ at the first follow-up visit with an event of CIN2+.

* The total clearance rate: (1) + (2) = 55.6%

** Type-specific persistence rate: (3)-1 + (3)-2 = 14.4%

[#]New acquisition rate: (3)-2 + (4)-1 + (4)-2 + (5)-1 + (5)-2 = 28.0%

Supplementary Table 11. 5-year, 10-year and 15-year cumulative recurrent CIN2+ rates according to cotest results after conization in the surveillance group (n = 425)

**Consecutive cotest results after conization**		**No. of patients**	**5-year cumulative recurrent CIN2+ rate (%)**	**10-year cumulative recurrent CIN2+ rate (%)**	**15-year cumulative recurrent CIN2+ rate (%)**
**HPV+/Cytology+ at first follow-up visit**		37	43.2	64.8	71.9
**HPV+/Cytology- at first follow-up visit**		128	10.2	18.7	25.0
**HPV-/Cytology+ at first follow-up visit**		4[a]	25.0	25.0	NA (max follow-up 13.3 years)
**HPV-/Cytology- at first follow-up visit**	1-negative cotest	62	1.7	1.7	17.3
	2 or 3-negative cotests	41	0 (no recurrence)	7.7 (1 recurrence at 6.6 years)	7.7
	≥4-negative cotests	153	0.8 (1 progression to IB1 at 3.1 years)[b]	0.8	0.8

CIN2+, cervical intraepithelial neoplasia grade 2 or worse; *HPV*, human papillomavirus.

[a] These 4 patients (C0102, C0137, C0139, C0202) had HPV- and cytology (dysplasia cannot rule out HSIL, ASCUS, ASCUS, ASCUS, respective) at the first follow-up. Patient C0102 (SP group) underwent a colposcopy directed cervical biopsy proven recurrent CIN3 (HPV16+) in 2005 at 5.9 months. She received a second conization (HPV-) and had a subsequent HPV-/cytology negative result in 2006. She was enrolled in 2009. She remained HPV16+/cytology- for 5 visits and staring to have cytology of

ASCUS in 2012, when her colposcopy was negative. Six months later, her cytology showed AGC favor neoplasm and cervical biospy showed adenocarcinoma IB1 (supplementary Table 6). The other two patients had acquired new low risk types but without events during follow-up, and the remaining one (C0139) remained both HPV-/cytology- without events.

[b] This patient (SN-C0326) had 5 consecutive negative cotests since first follow-up after initial conization, however HPV16+ reemerged at 31 months with normal Pap smear, when colposcopic directed cervical biopsy revealed CIN3 ruled out microinvasion. Conization revealed invasive squamous carcinoma, and the final pathology of the radical hysterectomy was stage IB1 (see also **Supplementary Table 12**).

Supplementary Figure 6. Cumulative recurrent CIN2+ rates according to cotest results after conization.

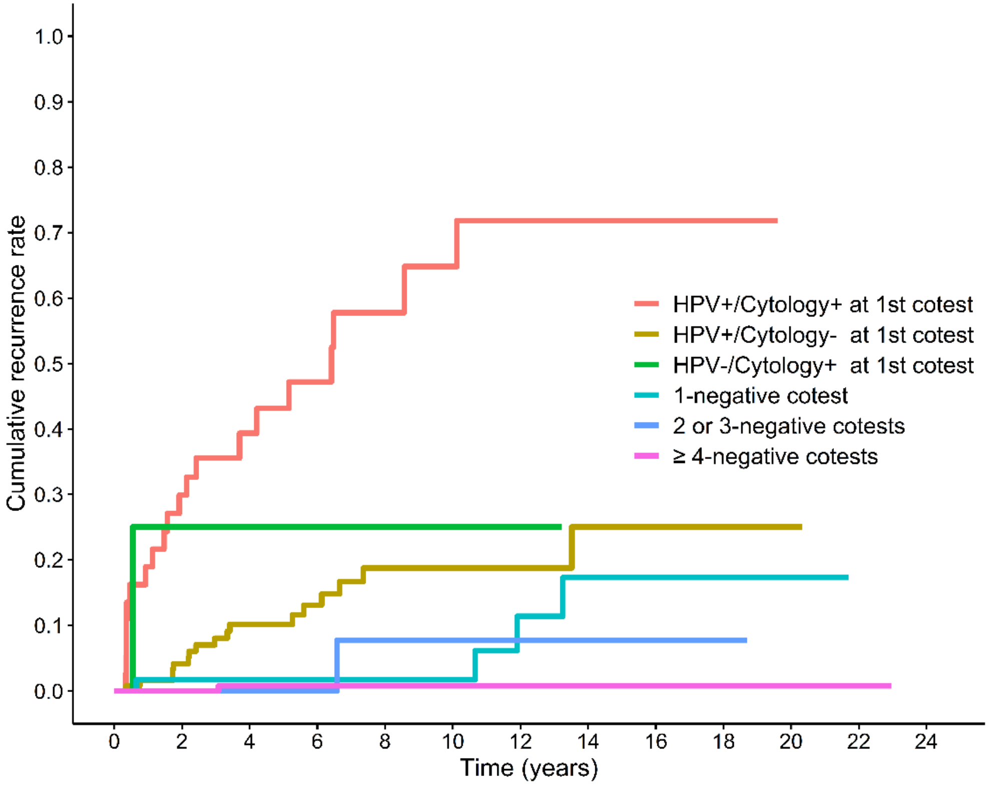

Number at risk

37	25	16	10	7	5	4	3	1	1	0	0	0
128	105	77	54	33	25	20	11	7	6	2	0	0
4	3	2	2	2	2	1	0	0	0	0	0	0
62	49	37	30	30	22	17	13	8	6	3	0	0
41	33	19	13	11	11	8	5	3	2	0	0	0
153	151	113	96	77	61	48	34	18	12	6	3	0

Supplementary Table 12. HPV reemergence[a] or acquisition[b] after 2 or ≥3 consecutive co-test negative results in the SN group

		Initial /Enrollment	Visit 1	Visit 2	Visit 3	Visit 4	Visit 5	Visit 6	Visit 7
SN-C0300	HPV	52,59	59	negative	negative	negative	74 [b]	74 [b]	
	Cytology/Histology	CIN3	normal	normal	normal	normal	normal	normal	normal
SN-C0310	HPV	18	negative	negative	32 [b],33 [b]	32			
	Cytology/Histology	CIN2	normal	normal	normal	normal			
SN-NV072	HPV	16	negative	negative	negative	negative	18 [b]	18,66 [b]	
	Cytology/Histology	CIN2	normal	normal	normal	normal	ASCUS/NPD	LSIL/colposcopy-	
SN-NV144	HPV	16	negative	negative	negative	53 [b]			
	Cytology/Histology	CIN3	normal	normal	normal	normal			
SN-C0326	HPV	16	negative	negative	negative	negative	negative	16[c]	
	Cytology/Histology	CIN3	normal	normal	normal	normal	normal	normal/IB1	
SN-C0359	HPV	52	negative	negative	negative	61 [b]	negative		
	Cytology/Histology	CIN2	normal	normal	normal	normal	normal		
SN-C0373	HPV	52	negative	negative	negative	negative	negative	53 [b]	negative
	Cytology/Histology	CIN3	normal	ASCUS	normal	normal	normal	normal	normal
SN-NV024	HPV	33,58	negative	negative	52 [b]	negative			
	Cytology/Histology	CIN2	normal	normal	normal	normal			
SN-NV300	HPV	58,59	negative	negative	74 [b]	74			

	Cytology/Histology	CIN2	normal	normal	normal	normal	normal		
SN-NV379	HPV	33	negative	negative	negative	56 [b]	negative		
	Cytology/Histology	CIN2	normal	normal	normal	normal	normal		
SN-NV462	HPV	16	negative	negative	negative	negative	71 [b]		
	Cytology/Histology	CIN3	normal	normal	normal	normal	normal		

[a]The previous positive type became negative on ≥2 visits but turned positive again.

[b]HPV acquisition was defined as detection of HPV following at least two preceding visits with HPV-negative visits.

[c]This patient had 5 consecutive cotests negative, but reemergence of HPV16 with normal Pap smear, when colposcopic directed cervical biopsy revealed CIN3 r/o microinvasion. Conization revealed invasive squamous carcinoma, and the final pathology of the radical hysterectomy was stage IB1.

Supplementary Table 13. HPV re-emergence[a] or acquisition[b] after 2 or ≥3 consecutive co-test negative results in the SP group

Patient code		Initial	Visit -6	Visit -5	Visit -4	Visit -3	Visit -2	Visit -1	Enrollment Visit1	Visit 2	Visit 3
SP-C0002	HPV	52	52	negative	70	58,70	70	70	70	negative	negative
	Cytology/Histology	CIN3	normal	normal	ASCUS/CIN1	normal	normal	normal	normal	normal	normal
SP-C0009	HPV	18,58	NA	NA	NA	58[a],62[b]	53[b],62	53	18[a],82	negative	negative
	Cytology/Histology	CIN2	NA	NA	NA	CIN3[d]	Normal	Normal	Normal	normal	normal
SP-C0011	HPV	16	NA	NA	NA	NA	NA	negative	negative	negative	negative
	Cytology/Histology	CIN3	NA	NA	NA	NA	NA	normal	normal	normal	normal
SP-C0016	HPV	16	NA	NA	NA	NA	16,84	39,81,84	39	39	39
	Cytology/Histology	CIN2	NA	NA	NA	NA	NA	HSILCIN1[d]	normal	CIN1[c]	normal
SP-C0018	HPV	16	NA	NA	NA	NA	NA	NA	negative	negative	negative
	Cytology/Histology	CIN3	normal	normal	normal	normal	normal	normal	normal	normal	normal
SP-C0029	HPV	16,18,31	NA	NA	NA	negative	negative	negative	negative	6[b]	6,54[b]
	Cytology/Histology	CIN2	NA	NA	NA	normal	normal	normal	normal	normal	normal
SP-C0031	HPV	16	NA	NA	NA	NA	NA	NA	62	62	negative
	Cytology/Histology	CIN3	NA	normal	normal	normal	normal	normal	normal	normal	normal
SP-C0048	HPV	58	NA	51,58	negative	51	51	51	51 [e]	negative	negative
	Cytology/Histology	CIN2	NA	normal	normal	normal	normal	normal	normal	normal	normal
SP-C0054	HPV	CIN2	NA	NA	NA	CIN3[d]	Normal	Normal	Normal	normal	normal
	Cytology/Histology	CIN2	normal	normal	normal	normal	normal	normal	normal	normal	normal

SP-C0058[f]	HPV	18[c]	negative	negative	84[b]	84	negative	negative	18[a]	18	18
	Cytology/Histology	CIN3	NA	normal	normal	normal	normal	normal	HSIL/CIN3[d]	CIN3d	SCC[g]
SP-C0061	HPV	16	NA	NA	NA	NA	NA	NA	16	33,52,84	52
	Cytology/Histology	CIN2	NA	NA	NA	NA	NA	NA	normal	normal	normal
SP-C0063	HPV	18,52	NA	NA	52	negative	negative	negative	negative	negative	negative
	Cytology/Histology	CIN2	NA	NA	normal	normal	normal	normal	normal	normal	normal
SP-C0064	HPV	16	NA	NA	negative	54	negative	54	54,66[b]	negative	negative
	Cytology/Histology	CIN3	normal	normal	normal	normal	normal	normal	normal	normal	normal
SP-C0074	HPV	16	NA	NA	NA	NA	NA	negative	negative	negative	negative
	Cytology/Histology	CIN3	normal	normal	normal	normal	normal	normal	normal	normal	normal
SP-C0079	HPV	16	negative	Negative	NA	84[b]	11[b]	negative	negative	negative	negative
	Cytology/Histology	CIN3	normal	normal	normal	normal	normal	normal	normal	normal	CIN1
SP-C0081	HPV	18,58	NA	NA	NA	NA	NA	NA	L1AE5	58,69,71,L1AE5	18
	Cytology/Histology	CIN2	NA	NA	NA	NA	NA	NA	normal	normal	CIN2[d]
SP-C0090	HPV	16	18	negative	39	39	negative	negative	negative	negative	negative
	Cytology/Histology	CIN2	normal	normal	normal	normal	normal	normal	normal	normal	normal
SP-C0112	HPV	82	negative	negative	NA	66[b]	negative	53[b],70[b],71[b]	44[b],61,70,71	70	32[b],51[b],70,71
	Cytology/Histology	CIN2	normal	normal	normal	normal	normal	normal	normal	normal	normal
SP-C0116	HPV	16	NA	negative	52	negative	negative	31[b]	negative	negative	negative

	Cytology/Histology	CIN2	normal	normal	normal	normal	normal	ASC-US	normal	normal	normal
SP-C0137	HPV	16	NA	negative	negative	53[b],54[b]	53,84	53,68,84[b]	53	53,61[b],62[b],74[b]	61,62,74
	Cytology/Histology	CIN2	normal	normal	normal	CIN1[c]	CIN1	normal	normal	normal	normal
SP-C0140	HPV	58	51,58	51,58	negative	negative	44[b].58[a], 66[b]	44	44	44,81[b]	44,53[b],81
	Cytology/Histology	CIN2	ASC-US	ASC-US	normal	normal	normal	normal	normal	normal	ASC-US
SP-C0194	HPV	33,42	negative	negative	44[b],53[b]	44,74[b]	negative	negative	74	74	negative
	Cytology/Histology	CIN2	normal	normal	normal	normal	normal	normal	normal	normal	normal
SP-C0197	HPV	52	negative	NA	negative	NA	NA	NA	negative	negative	55[b]
	Cytology/Histology	CIN3	normal	normal	normal	normal	normal	normal	normal	normal	normal
SP-C0199	HPV	39,58	negative	NA	NA	NA	negative	NA	negative	negative	negative
	Cytology/Histology	CIN2	normal	normal	normal	normal	normal	normal	normal	normal	normal
SP-C0202	HPV	51	NA	NA	NA	NA	NA	NA	negative	negative	negative
	Cytology/Histology	CIN2	NA	NA	NA	NA	NA	normal	normal	normal	normal
SP-C0215	HPV	53	NA	NA	NA	NA	NA	NA	negative	negative	negative
	Cytology/Histology	CIN2	normal	normal	normal	normal	normal	normal	normal	normal	normal
SP-C0231	HPV	16,18	NA	NA	NA	NA	NA	NA	negative	negative	62[b]
	Cytology/Histology	CIN2	NA	NA	normal	normal	normal	normal	normal	normal	normal
SP-C0306	HPV	16,31	NA	44,56,71	44,56,62,71	44,56,62,69,71	44,56,71	56,81	44,56	44,56	56
	Cytology/Histology	CIN2	normal	normal	CIN1	normal	normal	ASC-US	normal	normal	normal

SP-C0307	HPV	52	18,84	16,31	16,31,54	16,43,54,81	6,54,82	negative	negative	33[b]	33
	Cytology/Histology	CIN3	normal	normal	normal	normal	normal	normal	normal	normal	CIN1[d]
SP-C0348	HPV	51	NA	NA	NA	NA	NA	51	negative	negative	negative
	Cytology/Histology	CIN3	NA	NA	NA	NA	NA	normal	normal	normal	normal
SP-C0349	HPV	16	NA	66	55	45,55	negative	66	negative	negative	negative
	Cytology/Histology	CIN2	normal	normal	normal	normal	normal	normal	normal	normal	normal
SP-NV016	HPV	16,58	negative	negative	51[b]	58[a]	51,58	51,58	84[b]	58,84	59[b]
	Cytology/Histology	CIN2	normal	normal	negative	negative	ASC-US	CIN2[d]	normal	normal	normal
SP-NV056	HPV	33	NA	NA	negative	negative	NA	NA	81[b]	negative	negative
	Cytology/Histology	CIN2	NA	NA	normal	normal	normal	normal	normal	normal	normal
SP-NV073	HPV	82	NA	NA	NA	NA	NA	NA	82	NA	negative
	Cytology/Histology	CIN3	NA	NA	NA	NA	NA	normal	normal	normal	normal
SP-NV158	HPV	52	NA	NA	NA	NA	NA	NA	62	negative	negative
	Cytology/Histology	CIN2	NA	NA	normal	normal	normal	normal	normal	normal	normal
SP-NV173	HPV	16	NA	NA	NA	NA	NA	NA	negative	negative	negative
	Cytology/Histology	CIN3	NA	NA	NA	NA	NA	NA	normal	normal	normal
SP-NV214	HPV	58	NA	NA	NA	NA	NA	58	negative	negative	negative
	Cytology/Histology	CIN2	NA	NA	NA	NA	NA	normal	normal	normal	normal

Continued Supplementary Table 13. HPV re-emergence[a] or acquisition[b] after two or three cytology co-test negative results in the SP group

		Visit 4	Visit 5	Visit 6	Visit 7	Visit 8	Visit 9	Visit 10	Visit 11
SP-C0002	HPV	negative	62[b]	negative	18[b]	negative	negative	negative	
	Cytology/Histology	normal	normal	normal	normal /CIN1[c]	Normal	normal	normal	
SP-C0009	HPV	negative	negative	61[b]	61	61	negative	negative	61
	Cytology/Histology	normal	normal	normal	normal	Normal	normal	normal	normal
SP-C0011	HPV	32b	32	negative	NA	NA	NA	NA	NA
	Cytology/Histology	normal	normal	normal	NA	NA	NA	NA	NA
SP-C0016	HPV	39,81[b]	negative	negative	negative	70[b]	81	negative	negative
	Cytology/Histology	CIN1[c]	normal	normal	normal	CIN1[c]	normal	CIN1[c]	normal
SP-C0018	HPV	negative	70	56,70,81	negative	negative	negative	negative	70
	Cytology/Histology	normal	normal	normal	normal	Normal	normal	normal	normal
SP-C0029	HPV	6[b],54[b]	negative	negative	NA	NA	NA	NA	NA
	Cytology/Histology	normal	normal	normal	NA	NA	NA	NA	NA
SP-C0031	HPV	negative	negative	negative	negative	39[b]	39	negative	negative
	Cytology/Histology	normal	normal	normal	normal	Normal	normal	normal	normal
SP-C0048	HPV	negative	56[b]	18[b],56	56	56	18,42[b],56	56	negative
	Cytology/Histology	normal	normal	normal	normal	Normal	normal	CIN1[c]	normal
SP-C0054	HPV	58,69,72	58,72	58,69,72	58,72	72	72	69,72	69,72
	Cytology/Histology	normal	normal	normal	normal	normal	normal	normal	normal
SP-C0058	HPV	NA	NA	NA	NA	NA	NA	NA	NA

	Cytology/Histology	NA	NA	NA	NA	NA	NA	NA	NA
SP-C0061	HPV	52, 84	52	negative	negative	negative	negative	39[b]	39,54[b]
	Cytology/Histology	Normal	CIN2[d]	normal	normal	normal	normal	normal	normal
SP-C0063	HPV	negative	negative	negative	negative	negative	42[b]	negative	NA
	Cytology/Histology	normal	normal	normal	normal	normal	normal	normal	NA
SP-C0064	HPV	negative	6[b]	6	6	6	negative	negative	32[b]
	Cytology/Histology	normal	normal	CIN1[c]	CIN1[c]	negative	normal	normal	normal
SP-C0074	HPV	84[b]	negative	negative	negative	negative	18[b]	negative	negative
	Cytology/Histology	normal	normal	normal	normal	normal	normal	ASC-US	normal
SP-C0079	HPV	negative	42[b]	62[b]	62	62	negative	32[b]	NA
	Cytology/Histology	normal	normal	normal	normal	ASC-US	normal	CIN1	NA
SP-C0081	HPV	58,69,71,L1AE5	58,69,71,L1AE5	69	69	negative	negative	negative	L1AE5
	Cytology/Histology	normal	normal	CIN1[c]	normal	normal	normal	normal	normal
SP-C0090	HPV	negative	negative	negative	71[b]	71	71	negative	negative
	Cytology/Histology	normal	normal	normal	normal	normal	normal	normal	normal
SP-C0112	HPV	32,70,71	71	negative	negative	54[b],71,84[b]	84	70,71,84	84
	Cytology/Histology	normal	normal	normal	normal	normal	normal	normal	normal
SP-C0116	HPV	negative	negative	negative	negative	53[b]	53	negative	negative
	Cytology/Histology	normal	normal	normal	normal	normal	normal	normal	normal
SP-C0137	HPV	62,74	62	62,70	62,70[b]	62	62	NA	NA
	Cytology/Histology	normal	normal	normal	normal	normal	normal	NA	NA
SP-C0140	HPV	53[b]	53	negative	84[b]	negative	53,84	negative	53

	Cytology/Histology	normal	CIN1[c]	normal	normal	normal	normal	normal	normal
SP-C0194	HPV	negative	negative	74	negative	negative	negative	74	74
	Cytology/Histology	normal	normal	normal	normal	normal	normal	normal	normal
SP-C0197	HPV	negative	52[a]	negative	negative	negative	52[a]	NA	NA
	Cytology/Histology	normal	CIN1[c]	normal	normal	normal	normal	NA	NA
SP-C0199	HPV	31[b]	negative	negative	negative	68[b]	negative	negative	negative
	Cytology/Histology	normal	normal	normal	normal	normal	normal	normal	normal
SP-C0202	HPV	negative	44[b]	negative	NA	NA	NA	NA	NA
	Cytology/Histology	normal	normal	normal	NA	NA	NA	NA	NA
SP-C0215	HPV	51[b],74[b]	51,74	16[b],51,74	NA	NA	NA	NA	NA
	Cytology/Histology	normal	normal	normal	normal	NA	NA	NA	NA
SP-C0231	HPV	62	62	62,71	71	negative	71	71	71
	Cytology/Histology	normal	normal	normal	normal	normal	normal	normal	normal
SP-C0306	HPV	44,56	44,56	negative	negative	56[b]	44[b],56	56	56
	Cytology/Histology	normal	normal	normal	normal	normal	normal	normal	normal
SP-C0307	HPV	negative	negative	negative	53[b]	negative	negative	negative	negative
	Cytology/Histology	normal	normal	normal	normal	normal	normal	normal	normal
SP-C0348	HPV	negative	negative	11[b]	NA	NA	NA	NA	NA
	Cytology/Histology	normal	normal	normal	normal	normal	NA	NA	NA
SP-C0349	HPV	negative	33[b],52[b]	33,52	33	33	33	33,39[b]	negative
	Cytology/Histology	normal	normal	CIN1[c]	normal	CIN1 [c]	CIN1 [c]	normal	normal
SP-NV016	HPV	59,67	53,58,67	52,53,58	52,58,67	51,52	51,52,58,54	51,52	51

	Cytology/Histology	ASC-US	normal	normal	CIN1[c]	CIN1[c]	normal	normal	normal
SP-NV056	HPV	negative	81[b]	NA	NA	NA	NA	NA	NA
	Cytology/Histology	normal	normal	NA	NA	NA	NA	NA	NA
SP-NV073	HPV	negative	58[b]	NA	NA	NA	NA	NA	NA
	Cytology/Histology	normal	normal	normal	NA	NA	NA	NA	NA
SP-NV158	HPV	negative	NA	NA	NA	NA	negative	71[b]	NA
	Cytology/Histology	normal	normal	normal	normal	normal	normal	normal	NA
SP-NV173	HPV	51[b],56[b]	negative	negative	70[b]	70	70	negative	NA
	Cytology/Histology	normal	normal	normal	normal	normal	normal	normal	NA
SP-NV214	HPV	negative	NA	NA	negative	negative	62[b]	negative	negative
	Cytology/Histology	normal	normal	normal	normal	normal	normal	normal	normal

[a]The previous positive type became negative on ≥2 visits but turned positive again.

[b]HPV acquisition was defined as detection of HPV following at least two preceding visits with HPV-negative visits.

[c]These patients received laser therapy.

[d]These patients received conization.

[e]These patients received intravaginal imiquimod cream.

[f]This patient had HPV-18 CIN3 at 7 years before her enrollment who acquired HPV84 but subsequently cleared and had a recurrent CIN 3 at 7 months before enrollment and subsequent follow-up Pap smear was HSIL a hysterectomy was advised but declined by the patient. She was enrolled and HPV18+ with Pap SCC/biopsy CIN3 and treated with hysterectomy and final pathology revealed FIGO stage squamous cell carcinoma IA1.

[g]These patients received hysterectomy.

*Normal includes non-malignant diagnosis such as inflammation, squamous metaplasia, atrophy, erosion
